# Comparative genomic and methylome analysis of non-virulent D74 and virulent Nagasaki *Haemophilus parasuis* isolates

**DOI:** 10.1371/journal.pone.0205700

**Published:** 2018-11-01

**Authors:** Tracy L. Nicholson, Brian W. Brunelle, Darrell O. Bayles, David P. Alt, Sarah M. Shore

**Affiliations:** National Animal Disease Center, Agricultural Research Service, USDA, Ames, Iowa, United States of America; Tianjin University, CHINA

## Abstract

*Haemophilus parasuis* is a respiratory pathogen of swine and the etiological agent of Glässer's disease. *H*. *parasuis* isolates can exhibit different virulence capabilities ranging from lethal systemic disease to subclinical carriage. To identify genomic differences between phenotypically distinct strains, we obtained the closed whole-genome sequence annotation and genome-wide methylation patterns for the highly virulent Nagasaki strain and for the non-virulent D74 strain. Evaluation of the virulence-associated genes contained within the genomes of D74 and Nagasaki led to the discovery of a large number of toxin-antitoxin (TA) systems within both genomes. Five predicted hemolysins were identified as unique to Nagasaki and seven putative contact-dependent growth inhibition toxin proteins were identified only in strain D74. Assessment of all potential *vtaA* genes revealed thirteen present in the Nagasaki genome and three in the D74 genome. Subsequent evaluation of the predicted protein structure revealed that none of the D74 VtaA proteins contain a collagen triple helix repeat domain. Additionally, the predicted protein sequence for two D74 VtaA proteins is substantially longer than any predicted Nagasaki VtaA proteins. Fifteen methylation sequence motifs were identified in D74 and fourteen methylation sequence motifs were identified in Nagasaki using SMRT sequencing analysis. Only one of the methylation sequence motifs was observed in both strains indicative of the diversity between D74 and Nagasaki. Subsequent analysis also revealed diversity in the restriction-modification systems harbored by D74 and Nagasaki. The collective information reported in this study will aid in the development of vaccines and intervention strategies to decrease the prevalence and disease burden caused by *H*. *parasuis*.

## Introduction

*Haemophilus parasuis* is a small, Gram negative, non-motile, pleomorphic rod-shaped, and nicotinamide adenine dinucleotide (NAD)-dependent bacterium of the *Pasteurellaceae* family [1, 2]. *H*. *parasuis* is a respiratory pathogen affecting swine and is the etiological agent of Glässer's disease, a systemic infection resulting in arthritis, polyserositis (inflammation of serous membranes), and meningitis [2–4]. Additionally, *H*. *parasuis* infections can lead to pneumonia without signs of systemic disease in swine [5–7]. The morbidity and mortality caused by *H*. *parasuis* is a significant source of economic loss to the swine industry worldwide.

Serotyping based on the production of heat-stable antigens, in which capsular polysaccharide is presumed to be the dominant component of the serotyping antigen, is routinely used for isolate classification and epidemiological purposes as well as for guidance in regards to vaccination strategies. Fifteen serovars of *H*. *parasuis* have been defined, however, a substantial percentage of clinical isolates are identified as nontypeable (NT) using conventional indirect hemagglutination (IHA) methods [8, 9]. Progress to alleviate this problem has been made with the determination of the nucleotide sequence of the capsule locus from fifteen serovar reference isolates, which has been used to develop molecular serotyping methods [10–12].

*H*. *parasuis* isolates can exhibit different virulence capabilities ranging from lethal systemic disease to subclinical carriage. Numerous studies have focused on the identification of virulence factors that enable some isolates to cause systemic disease, distinguishing them from isolates that remain colonizers of the upper respiratory tract. Examples of potential virulence factors that have been evaluated to date include capsule production, outer membrane proteins (OMPs), trimeric autotransporters, and regulatory proteins QseC and OxyR [13–22]. Despite the advancement in our understanding of the pathogenic mechanisms used by *H*. *parasuis*, a direct link between specific virulence factors and the ability to cause systemic disease has not been demonstrated. Accordingly, virulence is thought to be multifactorial [2–4]. Data directly linking specific genes to disease outcomes has been hindered by several substantial complications, the most notable being difficulties in genetic modification of the chromosome due to low transformation efficiencies attributed to strain specific restriction modification barriers, as well as difficulties in consistently reproducing Glässer's disease in conventionally raised pigs due to confounding factors such as age, health status, differences in maternal antibody titers towards *H*. *parasuis*, and coinfection with other respiratory pathogens [23–26].

There are no effective approaches to eradicate *H*. *parasuis* from pig herds and controlling outbreaks has proven difficult [2, 27]. Although vaccines have been developed, most are comprised of bacterins, resulting in poor heterologous protection. Consequently no broadly protective vaccines or intervention strategies exist [28–30]. The current treatment for *H*. *parasuis* is broad spectrum antibiotics, which are expensive and are believed to increase the risk of resistant strain development [29, 31–33]. Additionally, with increased pressure to limit antibiotic use in agriculture, alternative approaches are desperately needed to reduce disease burden and economic losses caused by *H*. *parasuis*.

In a previous effort to link genomic differences to disease outcome, draft genome sequence data was obtained for ten genetically distinct isolates along with the evaluation of virulence in Caesarean-derived, colostrum deprived (CDCD) pigs [34]. These results demonstrated that strain D74 is a non-virulent colonizer of the upper respiratory tract, while in contrast, strain Nagasaki was highly virulent and capable of causing systemic disease [34]. Many genomic differences, including gene content and/or nucleotide variation, were identified that could account for the phenotypic difference between the strains [34]. Unfortunately many genes or regions of interest within each strain were incomplete, preventing a reliable one-to-one assignment and subsequent comparison of any predicted protein structure. In order to definitively identify and characterize the genomic differences between the highly virulent Nagasaki strain and the non-virulent D74 strain, the goal of our report was to obtain the closed whole-genome sequence and genome-wide methylation patterns between these phenotypically distinct strains.

## Materials and methods

### Genome sequencing and annotation

*H*. *parasuis* strain Nagasaki is a Serotype Type 5 reference Strain and a Multilocus sequence typing (MLST) Type 24 strain. *H*. *parasuis* strain D74 is a Serotype Type 9 reference Strain and a MLST Type 25 strain. Strains were cultured in Brain Heart Infusion (BHI) Broth (BD Biosciences, Sparks, MD) supplemented with 5% filtered heat-inactivated horse serum (GIBCO, Life Technologies, Grand Island, NY) and 0.01% (w/v) nicotinamide adenine dinucleotide (NAD) (Sigma-Aldrich, St. Louis, MO) at 37°C in 5% CO_2_ for 24 hours and total genomic DNA was extracted using the High Pure PCR Template Preparation Kit (Roche Applied Science, Indianapolis, IN). Whole genome sequencing was performed using both the Pacific Biosciences (PacBio) and Illumina MiSeq platforms. Library preparation for PacBio sequencing was performed following the PacBio 10-kb insert library preparation protocol available online at (http://www.pacb.com/wp-content/uploads/2015/09/Procedure-Checklist-10-kb-Template-Preparation-and-Sequencing.pdf). The 10 kb library for each strain was sequenced using the PacBio RSII platform with two SMRT cells for each isolate. Indexed libraries for the MiSeq protocol were generated with the Nextera XT DNA sample preparation and index kits (Illumina, San Diego, CA), pooled, and sequenced using MiSeq v2 500-Cycle reagent kit yielding 2 x 250-bp paired-end reads (Illumina, San Diego, CA).

Whole genome assemblies were generated using the PacBio smrtanalysis v. 2.3.0 (https://www.pacb.com/products-and-services/analytical-software/smrt-analysis/) and CANU v. 1.3 [35] software. The average PacBio coverage for the assembled genomes was 805x for Nagasaki and 1,284x for D74. Assembling the PacBio data for each strain resulted in a fully sequenced closed circular chromosome, which was subsequently oriented to start at the *dnaA* gene and trimmed by removing any overlapping sequence. The genomes were then polished and error corrected using the Broad Institute’s Pilon v 1.18 [36] and Illumina data 103x and 120x average coverage for Nagasaki and D74, respectively. The closed genome for each strain was then annotated using NCBI's Prokaryotic Genome Annotation Pipeline (PGAP) and additional curation was performed using the Prokka annotation software (version 1.12) [37] along with a *Haemophilus*-specific custom database. To compare putative protein sequences between Nagasaki and D74, the RAST prokaryotic genome annotation server [38] (http://rast.nmpdr.org/) was used to map annotated protein coding sequences (CDS) to functional subsystems and performed a one-to-one BLASTP comparison between the strains. Genome organization was evaluated using the Artemis Comparison Tool [39] and Mauve [40].

### Plasmid sequencing and annotation

Assembled PacBio data for pD74 did not initially result in a circularized plasmid sequence. The complete nucleotide sequence was subsequently determined using a primer walking strategy employing five separate plasmid preparations isolated from strain D74 using a Wizard Plus SV Minipreps DNA Purification System (Promega, Madison, WI) according to the manufacturer's protocol. The resulting circularized plasmid sequence was then polished and error corrected using the Broad Institute’s Pilon v 1.18 [36] and Illumina data 104x average coverage for strain D74. The assembled pD74 sequence was then annotated using NCBI's Prokaryotic Genome Annotation Pipeline (PGAP). Further sequence analysis was carried out using BLASTN, BLASTX, and Tandem Repeats Finder [41].

### IS and CRISPR analysis

Genomes were submitted to ISfinder (https://www-is.biotoul.fr/) [42] for the identification of bacterial IS elements using default parameters. Genomes were submitted to CRISPRFinder (http://crispr.i2bc.paris-saclay.fr/Server/) [43] for the identification CRISPR elements using default parameters.

### Phenotypic analysis

Phenotypic antibiotic resistance was determined using the broth microdilution method by Iowa State University Veterinary Diagnostic Laboratory following standard operating procedures. Each isolate was tested using the Trek BOPO6F plate (Thermo Fisher Scientific Inc., Oakwood Village, OH) and minimum inhibitory concentrations (MICs) were determined. MICs were evaluated in accordance with Clinical Laboratory Standards Institute (CLSI) recommendations for resistance interpretations.

### Genomic antimicrobial resistance (AMR) analysis

ResFinder 2.1 from the Center for Genomic Epidemiology (http://www.genomicepidemiology.org/) and the Comprehensive Antibiotic Resistance Database (CARD) (https://card.mcmaster.ca/home) were employed for AMR determinant identification. Genomes submitted to ResFinder 2.1 were evaluated for AMR determinants using starting parameters of a threshold ID of 90% and a minimum length of 60% and final parameters of a threshold ID of 30% and a minimum length of 20%. Genomes submitted to CARD were evaluated for AMR determinants using the criteria “default–perfect and strict hits only”.

### Capsular loci analysis

The nucleotide sequences of genes within the capsule loci of Nagasaki and D74 reported by Howell et al. [10] were obtained from NCBI. Alignments of individual gene and protein sequences, as well as calculation of percent identities, were performed using the Geneious Alignment tool in Geneious 10.1.3 (Biomatters Ltd., Auckland, New Zealand). Global alignment with free end gaps parameters were used for both nucleotide and protein sequence alignments followed by determining the percent identity relative to Howell et al. [10]. Nucleotide and amino acid insertions are reported using HGVS nomenclature [44].

### *cdiA* gene analysis

The nucleotide sequences of genes within the *cdiA* region of D74 were evaluated using BLASTX. Translated coding sequences were extracted and evaluated for the occurrence of any domain using the Pfam 31.0 database (https://pfam.xfam.org/) [45].

### *vtaA* gene analysis

To ensure that all potential *vtaA* genes were identified in both Nagasaki and D74 genome annotations, the translated coding sequences were extracted and batch queried using the Pfam 31.0 database (https://pfam.xfam.org/) [45] for all occurrences of a YadA_anchor domain (PF03895) [15]. Geneious 10.1.3 (Biomatters Ltd., Auckland, New Zealand) was employed to evaluate and compare the genome location of *vtaA* genes in both Nagasaki and D74, including flanking upstream and downstream genes. The domain architecture and content of each *vtaA* gene from both Nagasaki and D74 was further evaluated for the occurrence of any domains using the Pfam 31.0 database (https://pfam.xfam.org/) [45]. BLASTN was employed to search sequences upstream of *vtaA* genes in both Nagasaki and D74 for the occurrence of the Nagasaki *vtaA4* promoter sequence identified by Pina et al. [15].

### Methylation analysis

Detection of modified bases (m6A, m4C, m5C) and clustering of modified sites to identify methylation associated motifs was performed using the RS_Modification_and_Motif_analysis.1 tool from the SMRT analysis package version 2.3.0. Briefly, raw reads were aligned to the complete genomes of D74 and Nagasaki and interpulse duration (IPD) ratios were measured for all pulses aligned to each position in the reference sequence (http://www.pacb.com/pdf/TN_Detecting_DNA_Base_Modifications.pdf). [46]

### SSR analysis

The nucleotide sequences of all putative RM genes identified in D74 and Nagasaki were evaluated for the occurrence of SSRs, tandem repeats or homopolymeric tracts of consisting of five or more bases, within the coding region and in the region encompassing 150 bp upstream of the putative start codon using Geneious 10.1.3 (Biomatters Ltd., Auckland, New Zealand).

### Accession number(s)

The whole-genome sequences for these isolates were deposited in DDBJ/ENA/GenBank with the accession numbers CP018034 for Nagasaki and CP018032 for D74 genome and CP18033 for the plasmid sequence. The sequence data, target sequences and associated details for methylation enzymes, used for analyses in this report have been deposited in the REBASE database (www.rebase.neb.com) [47]. RM-system and methylation motifs for both strains can be accessed via the index of the REBASE database (http://tools.neb.com/genomes/) or directly via this link: http://rebase.neb.com/cgi-bin/pacbioget?20940 for D74 or http://rebase.neb.com/cgi-bin/pacbioget?20939 for Nagasaki.

## Results and discussion

### Genome features of *H*. *parasuis* strains D74 and Nagasaki

The complete genome assembly and annotation of *H*. *parasuis* strain Nagasaki contains a single circular chromosome 2,348,962 base pairs (bp) in length, encodes a total of 2,268 predicted protein coding sequences (CDSs), and a G+C content of 40.0% (Table 1). Of the 2,268 CDSs, 95 were predicted to be pseudogenes. The complete genome assembly and annotation of *H*. *parasuis* strain D74 encompasses a single circular chromosome 2,467,568 base pairs (bp) in length, a total of 2,252 total predicted protein coding sequences (CDSs), and a G+C content of 39.7% (Table 1). Similar to Nagasaki, 95 out of the 2,252 CDSs were predicted to be pseudogenes. Despite the equivalent number, none of the predicted pseudogenes were similar between the stains (S1 Table and S2 Table). The difference in rRNA numbers between D74 and Nagasaki is consistent with other closed *H*. *parasuis* genomes available in GenBank (Table 1). The genomes of *H*. *parasuis* Nagasaki and D74 were assessed using PHASTER (http://phaster.ca/) [48] for the occurrence of phage regions. Nine phage regions, seven intact and two incomplete, were identified along the Nagasaki chromosome (Table 2). Six phage regions, one intact and five incomplete, were also identified along the D74 chromosome (Table 1). The phage regions identified are unique to each genome. The chromosomal location, including start and end nucleotide positions, length of phage region, and classification of phage regions for both strains are summarized in Table 2.

**Table 1:** General features of the genomes of *H*. *parasuis* strains D74 and Nagasaki.

	D74	Nagasaki
Sequence Type (ST)	9	5
**Chromosome Size (bp)**	2,467,568	2,348,962
G + C Content (%)	39.7%	40.0%
**Total CDSs**	2,252	2,268
Pseudogenes^a^	95	95
**Functional CDSs**	2,157	2,173
rRNA (16S-23S-5S)	6-6-7	6-6-8
**tRNA**	57	60
Phage Regions	6	9
Plasmid	1	0

^a^Encoding either an incomplete predicted protein sequence, a frameshift, or an internal stop codon.

**Table 2:** Phage regions identified in *H*. *parasuis* strains D74 and Nagasaki.

Strain	Region #	Start^a^	End^a^	Length^b^	Classification^c^
Nagasaki	1	75,146	95,579	20,434	Incomplete
	2	578,665	613,918	35.254	Intact
	3	930,933	982,262	51.330	Intact
	4	978,209	1,022,173	43,965	Intact
	5	1,146,622	1,187,091	40,470	Intact
	6	1,837,359	1,870,441	33,083	Intact
	7	1,890,250	1,901,023	10,774	Incomplete
	8	2,053,720	2,085,576	31,857	Intact
	9	2,113,966	2,153,632	39,667	Intact
D74	1	1,021,542	1,030,153	8,612	Incomplete
	2	1,179,090	1,197,266	18,177	Incomplete
	3	1,186,455	1,216,786	30,332	Incomplete
	4	1,303,971	1,339,245	35,275	Intact
	5	1,390,803	1,407,651	16,840	Incomplete
	6	1,868,288	1,889,812	21,525	Incomplete

^a^Basepair chromosomal location.

^b^Length of region depicted in base pairs.

^c^Classification determined by PHASTER analysis (http://phaster.ca/) [48].

**Fig 1:**
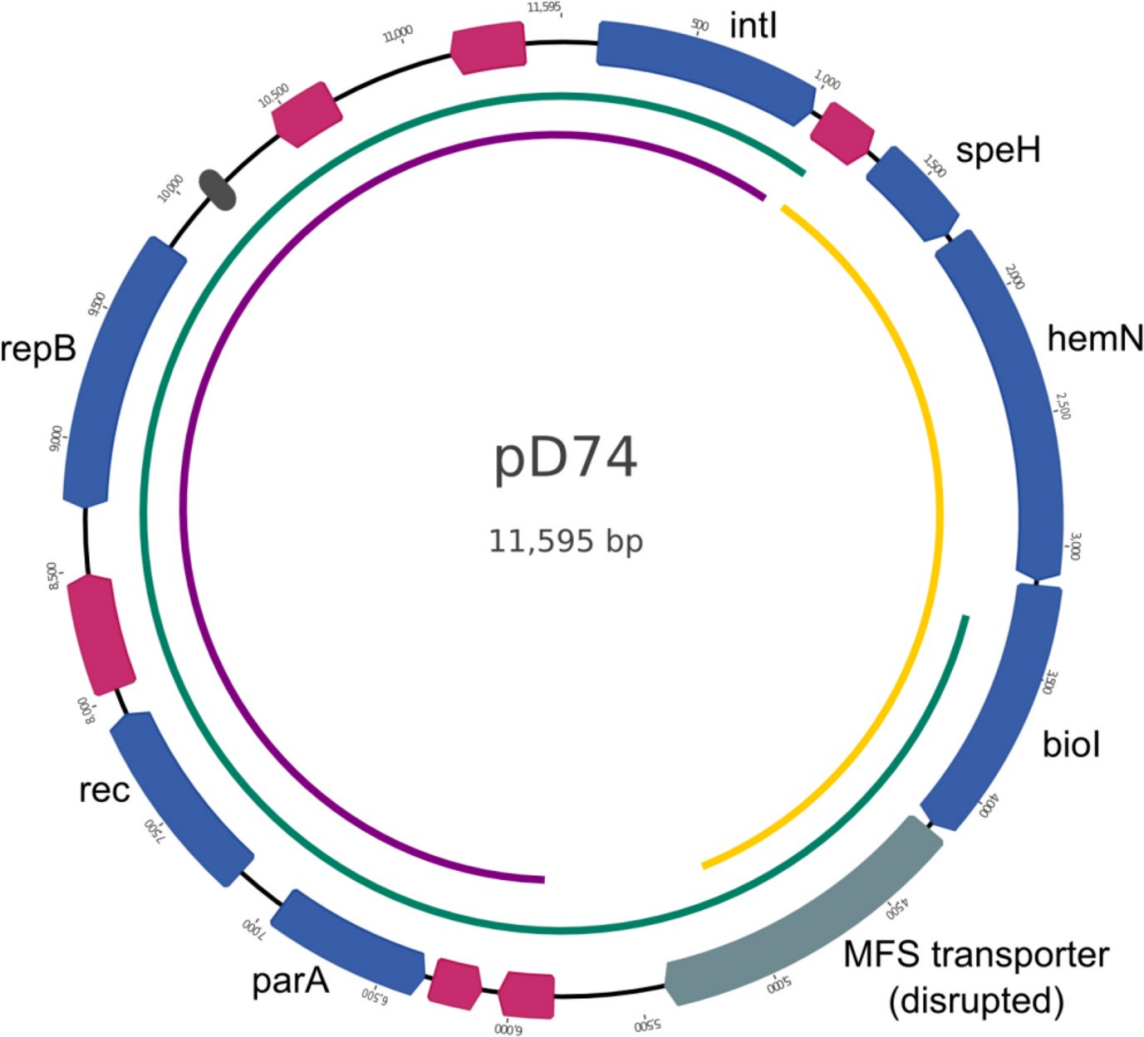
Map of plasmid pD74. Arrows indicate annotated CDSs; blue arrows represent CDSs with predicted functions based on sequence homology, pink arrows represent predicted CDSs of unknown function. The MFS transporter gene, labeled with a blue-grey arrow, is predicted to be a pseudogene. Dark grey box indicates region containing 4 copies of a 22-bp tandem repeat sequence identified using the Tandem Repeats Finder tool [41]. Arcs inside map indicate sequence with similarity to other sources; green arc represents sequence similar to *H*. *parasuis* plasmid pHS-Rec [49], purple arc represents sequence similar to a portion of the *Pasteurella trehalosi* plasmid pCCK13698 [50], and yellow arc represents sequence similar to a region from the genome sequence of *Gallibacterium anantis* strain UMN179 [51].

An 11,595-bp circular plasmid was additionally identified in the complete genome assembly and annotation of *H*. *parasuis* strain D74. With the addition of the plasmid, the total nucleotides, including both the circular chromosome and plasmid, is 2,479,163 bp for strain D74. The plasmid harbored by strain D74, designated as pD74, contains seven CDSs with predicted functions based on sequence homology, including *parA*, *rec*, and *repB*, which have predicted functions in plasmid replication. Additionally, pD74 harbors six CDSs of unknown function and one predicted pseudogene (Fig 1). These CDSs lacked a reciprocal match in Nagasaki. A region containing four copies of a 22-bp tandem repeat sequence was identified upstream of the *repB* CDS, which could potentially comprise the origin of replication (Fig 1). The sequence spanning from 3,328 to 1,243 bp comprises the entire 9,462 bp sequence of *H*. *parasuis* plasmid pHS-Rec (accession no. AY862436) with 99.2% sequence identity (Fig 1) [49]. The sequence spanning from 5,847 to 1,025 bp shares 99.4% sequence identity with *Pasteurella trehalosi* plasmid pCCK13698 (accession no. AM183225) (Fig 1) [50]. pD74 sequencing from 1,246 to 5,350 bp shares 73.9% sequence identity with a chromosomal region of *Gallibacterium anantis* strain UMN179 (accession no. CP002667) (Fig 1) [51].

### Comparison of the genome sequences of *H*. *parasuis* strains D74 and Nagasaki

**Fig 2:**
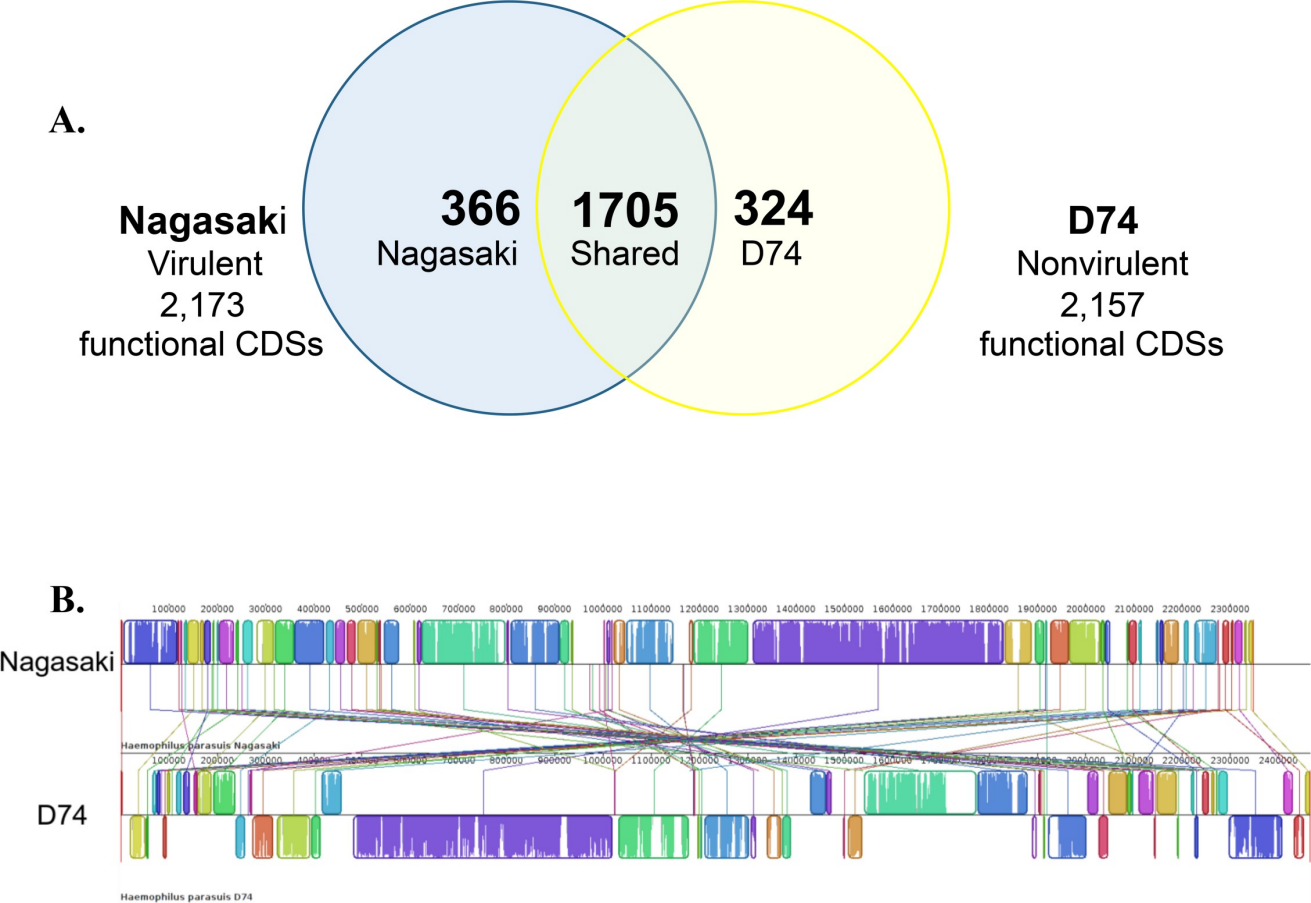
Comparison of the genome sequences of *H*. *parasuis* strains D74 and Nagasaki. (A) Distribution of protein-encoding genes in *H*. *parasuis* strains Nagasaki and D74. Venn diagram demonstrating the unique 2,173 protein-encoding genes in Nagasaki (blue), the 2,157 protein-encoding genes D74 (yellow), and the shared coding sequences defined as bi-directional best hits (center circle, green) via 1-to-1 reciprocal BLASTP comparison using RAST [38] (excluding frame-shifted genes). (B) Comparison of the linear organization of the *H*. *parasuis* strains Nagasaki and D74 chromosomes. Mauve [40] was used to compare the chromosomes of Nagasaki and D74. Locally collinear blocks (LCBs) representing regions of sequence that align in each genome are illustrated as colored rectangles connected by lines. Nagasaki was used as the reference sequence. In D74, LCBs placed above the center line are in the same orientation as in Nagasaki and LCBs placed below the center line are in the reverse orientation relative to Nagasaki. Blank sections are regions that did not align and are likely to contain unique or strain-specific sequence. Many of the larger blank regions not in LCBs contain loci predicted to encode products of phage origin.

A reciprocal or one-to-one BLASTP comparison of the protein coding sequences in Nagasaki and D74 identified 1,705 shared CDSs between the strains (Fig 2). Of the 2,173 functional CDSs in strain Nagasaki, 366 CDSs lacked a reciprocal match in D74 and were designated unique or Nagasaki-specific (Fig 2A). Conversely, 324 CDSs, out of the 2,157 functional CDSs in strain D74, lacked a reciprocal match in Nagasaki and were designated unique or D74-specific (Fig 2A). Comparison of the linear organization of the genomes of Nagasaki and D74 revealed many genome re-arrangements and inversions (Fig 2B). The alignment additionally resulted in 73 locally collinear blocks (LCBs), the largest of which is 520,607 bp (Fig 2B).

The genomes of *H*. *parasuis* Nagasaki and D74 were assessed for the presence of insertion sequence (IS) elements using ISfinder (https://www-is.biotoul.fr/) [42]. IS elements found previously in other *H*. *parasuis* and *Pasteurellaceae* genomes were identified in both D74 and Nagasaki genomes and these IS elements contained the greatest similarity to sequences from the IS*1595* family. Specifically, four IS*Hps3* and three IS*Hps4* IS elements were identified in D74, while one IS*Hps3* and four IS*Hps4* IS elements were identified in Nagasaki. *In silico* analysis revealed the occurrence of additional transposases annotated as belonging to other families, such as IS*256* and IS*110*, in both D74 and Nagasaki genomes that were not identified by ISfinder analysis, given that it typically does not identify frameshifted or truncated IS units. These transposases were annotated as pseudogenes and therefore are likely nonfunctional. Of the five complete IS units identified in Nagasaki, two are within regions of conserved sequence order and have homologs in D74. Three of the intact IS units identified in Nagasaki are located at the border of sequence rearrangement, with one located at the border of one of the phage regions. Six complete IS units were identified in D74 with two located at the border of rearranged regions. Of the four IS units that are present within regions of conserved sequence order, two are within regions of conserved sequence order and have homologs in Nagasaki and two are small sequence insertions located within a conserved region in which only the IS unit is the only sequence not conserved within the region. Clusters of regularly interspaced short palindromic repeats (CRISPR) and spacer sequences in the genomes of both D74 and Nagasaki were screened using CRISPRFinder (http://crispr.i2bc.paris-saclay.fr) [43]. No evidence for CRISPR sequences were found in either genome.

Phenotypic antibiotic resistance was determined for *H*. *parasuis* D74 and Nagasaki and results are summarized in S3 Table. Nagasaki exhibited phenotypic resistance to clindamycin while D74 exhibited “intermediate” limited phenotypic resistance to clindamycin; no other resistance was identified. The genomes of *H*. *parasuis* Nagasaki and D74 were screened for the presence of acquired resistance genes and known chromosomal mutations conferring clindamycin resistance. No *erm*, *lnu*, or other known resistance determinants were identified in either genome.

### Capsule loci in *H*. *parasuis* strains D74 and Nagasaki

Due to the importance of capsular polysaccharide in serotyping or strain classification, virulence, and vaccine related research areas, the CDSs located within the capsule loci of Nagasaki and D74 were compared to nucleotide sequences reported by Howell et al. [10]. Several nucleotide and amino acid differences were identified and are summarized in S4 Table and S5 Table. Eleven out of sixteen predicted proteins within the D74 capsule locus were found to contain amino acid differences compared to the sequences reported by Howell et al. [10]. The genes harboring the most or more noteworthy changes for D74 include *wzx2*, *astA*, *gltL*, and *wzs* (S4 Table). The predicted amino acid changes in AstA relative to the Howell et al. [10] sequence included a two amino acid insertion and are: N98_H99insHId, Q134R, S157A, V177I, V183I, I189V, S195N, and V2056M. In contrast, the predicted amino acid sequence for GltL is shorter than that reported in Howell et al. [10] due to a different predicted start codon. The predicted amino acid changes in GltL are M1_K8del and V9M. The predicted amino acid changes in Wzx2 relative to the Howell et al. [10] sequence are: V176A, I180V, A187V, S192N, R278H, P291S, and R373S (S4 Table). The predicted amino acid changes in Wzs relative to the Howell et al. [10] sequence are: E3K, L27I, V230A, V613A, and V622A (S4 Table).

In *H*. *parasuis* Nagasaki, twelve out of fourteen predicted proteins within the capsule locus were found to contain amino acid differences compared to the sequences reported by Howell et al. [10]. The genes harboring the more notable changes for Nagasaki include *funA*, *wcfQ*, and *wbgX* (S5 Table). FunA is predicted to result in a longer protein sequence compared to Howell et al. [10] due to a different predicted start codon and five amino acid changes within the shared region, along with 37 additional amino acids on the N-terminus. The predicted amino acid changes in FunA are M1ext-37, A2V, A6V, D27V, S28N, and V138I (S5 Table). The predicted amino acid changes in WcfQ relative to the Howell et al. [10] sequence are: E43K, I58V, S61T, I64T, F106S, D131G, F132S, N194K, Y262C, R269N, F270N, and L271F (S5 Table). WbgX is predicted to encode a shorter protein sequence compared to Howell et al. [10] due to a different predicted start codon. The predicted amino acid changes in WbgX are: M1del, K2M, E3N, F4Y, T177A, S343N, P344A, and A349I (S5 Table).

### Virulence-associated genes identified in *H*. *parasuis* strains D74 and Nagasaki

To identify factors that could support host colonization and/ or virulence, the D74 and Nagasaki annotations were searched for CDSs encoding predicted functions in adhesion, hemolysis, secretion, toxin production, or other virulence-associated roles. Twenty-one CDSs encoding predicted adhesins were identified in D74 including five outer membrane genes (*ompA*, *ompP1*, *ompP2*, *ompP5*, and *ompD15*), two *fimD* fimbrial usher genes located adjacent to each other along the chromosome, pertactin family virulence factor *aidA*, filamentous hemagglutinin transporter *fhaC*, adhesin autotransporter *bmaC*, and two type IV pili genes (Table 3). Corresponding orthologs for *pilT* and *bmaC* were not found in Nagasaki. Gene *leuC* (A2U21_06460) in Nagasaki was the uni-directional best match to *pilT* (A2U20_03770) in D74 with 56% global nucleotide sequence identity and gene *aidA2* (A2U21_04065) in Nagasaki was the uni-directional best match to *bmaC* (A2U20_08910) in D74 with 51% global nucleotide sequence identity (Table 3). Eighteen CDSs encoding predicted adhesins were identified in Nagasaki including five outer membrane genes (*ompA*, *ompP1*, *ompP2*, *ompP5*, and *ompD15*), one *fimD* fimbrial usher gene (chromosomally adjacent to a predicted pseudogene *fimB*), two pertactin family virulence factor genes *aidA* and *aidA2*, and two type IV pili (Table 4). *In silico* analysis of the region in D74 containing the two *fimD* genes compared to the *fimD* gene in Nagasaki indicated that the D74 *fimD2* (A2U20_08925) aligns with the 5’ end of the Nagasaki *fimD* (A2U21_03995) and the D74 *fimD* (A2U20_08920) aligns with the 3’ end of the Nagasaki *fimD* gene. This suggests that the two *fimD* genes in D74 could have potentially arisen from a frameshift within a single gene. A 51% sequence identity was observed between corresponding orthologs *esiB* (A2U20_01865) in D74 and *esiB2* (A2U21_08055) in Nagasaki (Table 3 and Table 4).

**Table 3:** Predicted virulence-associated genes identified in *H*. *parasuis* D74.

Group	D74 locus_tag	D74 Name	Product/ Function	Hit^a^	Nagasaki locus_tag	% Identity^b^
Adhesin	A2U20_02695	*pilW*	Type IV pilus biogenesis/stability protein PilW	bi	A2U21_08925	100
	A2U20_03215	*pulG*	Type II secretory pathway, pseudopilin PulG	bi	A2U21_08415	97
	A2U20_03220	*pulJ*	Type II secretory pathway, component PulJ	bi	A2U21_08410	96
	A2U20_03425	*pilM*	Type IV pilus biogenesis protein PilM	bi	A2U21_08215	98
	A2U20_03430	*pilN*	Type IV pilus biogenesis protein PilN	bi	A2U21_08210	98
	A2U20_03445	*pilQ*	Type IV pilus biogenesis PilQ	bi	A2U21_08195	94
	A2U20_03490	*ompP5*	Outer membrane protein P5	bi	A2U21_08150	92
	A2U20_03710	*ompP1*	Outer membrane protein precursor P1	bi	A2U21_07950	90
	A2U20_03770	*pilT*	pilT domain-containing protein	uni	A2U21_06460	56
	A2U20_04380	*ompP2*	Outer membrane protein P2 precursor	bi	A2U21_07315	85
	A2U20_04655	*ompD15*	Surface antigen (D15), outer membrane protein	bi	A2U21_07040	99
	A2U20_04855	*ompA*	Outer membrane protein A	bi	A2U21_06820	98
	A2U20_06825	*fhaC*	Filamentous hemagglutinin transporter protein FhaC	uni	A2U21_11135	52
	A2U20_07210	*pilA*	Type IV pilin PilA	bi	A2U21_10860	87
	A2U20_07215	*pilB*	Type IV fimbrial assembly ATPase PilB	bi	A2U21_10855	98
	A2U20_07220	*pilC*	Type IV fimbrial assembly protein PilC	bi	A2U21_10850	99
	A2U20_07225	*pilD*	Tfp pilus assembly pathway, fimbrial leader peptidase	bi	A2U21_10845	94
	A2U20_08340	*aidA*	Type V secretory pathway, adhesin AidA	bi	A2U21_04065	72
	A2U20_08910	*bmaC*	Adhesin BmaC autotransporter	uni	A2U21_04065	51
	A2U20_08920	*fimD*	Putative F17-like fimbrial usher	uni	A2U21_03995	98
	A2U20_08925	*fimD2*	Putative F17-like fimbrial usher	bi	A2U21_03995	99
Hemolysin	A2U20_02515	*prtC*	Serralysin C	uni	A2U21_00470	58
	A2U20_06885		Hemagglutinin/hemolysin-related protein	-		
	A2U20_07380	*hlyD*	Hemolysin secretion protein D	-		
	A2U20_08300	*shlB*	Hemolysin transporter protein ShlB	bi	A2U21_11135	53
	A2U20_08375	*osmY*	Osmotically-inducible protein OsmY; putative hemolysin	bi	A2U21_04100	100
	A2U20_09890	*ahpA*	Hemolysin regulation protein AhpA	bi	A2U21_02620	99
	A2U20_10965	*prtB*	Serralysin B, hemolysin-type calcium-binding region	uni	A2U21_00470	56
	A2U20_10970	*prtB2*	Serralysin B, hemolysin-type calcium-binding region	bi	A2U21_00470	69
Secretion	A2U20_01865	*esiB*	Putative secretory immunoglobulin A-binding protein	bi	A2U21_08055	51
	A2U20_09675		Putative periplasmic/secreted protein	bi	A2U21_02375	93
Toxin	A2U20_00255	*ebgC*	EbgC protein_Toxin-antitoxin biofilm protein TabA	bi	A2U21_00620	98
	A2U20_01495	*higA*	Antitoxin HigA_mRNA interferase antitoxin	uni	A2U21_00995	63
	A2U20_01500	*hicA*	Addiction module toxin HicA	uni	A2U21_01000	59
	A2U20_01850	*vapC*	VapC toxin family PIN domain ribonuclease	bi	A2U21_10000	78
	A2U20_02115	*hipA*	Serine/threonine-protein kinase toxin HipA	bi	A2U21_09500	95
	A2U20_02705	*higA2*	Antitoxin HigA_mRNA interferase antitoxin	bi	A2U21_08915	93
	A2U20_02710	*higB*	mRNA interferase toxin HigB	bi	A2U21_08910	96
	A2U20_02805	*tdeA*	Putative toxin and drug export protein A	bi	A2U21_08785	97
	A2U20_02840	*relE*	Addiction module toxin RelE	bi	A2U21_03035	59
	A2U20_03250	*mazF*	Programmed cell death toxin MazF	uni	A2U21_06190	52
	A2U20_03635	*hicA2*	Aaddiction module toxin HicA	bi	A2U21_02950	94
	A2U20_04570	*higA3*	Antitoxin HigA_mRNA interferase antitoxin	uni	A2U21_00995	63
	A2U20_04795	*pezT*	Antitoxin/toxin system zeta toxin	uni	A2U21_03130	53
	A2U20_04825	*cdtC*	Cytolethal distending toxin subunit CdtC	bi	A2U21_06570	99
	A2U20_04830	*cdtB*	Cytolethal distending toxin subunit CdtB	uni	A2U21_06565	95
	A2U20_04835	*cdtA*	Cytolethal distending toxin subunit CdtA	bi	A2U21_06560	99
	A2U20_04880	*higB2*	mRNA interferase toxin HigB	uni	A2U21_05325	55
	A2U20_04885	*higA4*	Antitoxin HigA_mRNA interferase antitoxin	uni	A2U21_08915	56
	A2U20_05030	*higA5*	Antitoxin HigA_mRNA interferase antitoxin	bi	A2U21_06130	98
	A2U20_05035	*higB3*	mRNA interferase toxin HigB	bi	A2U21_06135	99
	A2U20_05120	*cdtC2*	Cytolethal distending toxin subunit CdtC	uni	A2U21_06570	98
	A2U20_05125	*cdtB2*	Cytolethal distending toxin subunit CdtB	bi	A2U21_06565	96
	A2U20_05130	*cdtA2*	Cytolethal distending toxin subunit CdtA	uni	A2U21_06560	99
	A2U20_05385	*higA6*	Antitoxin HigA_mRNA interferase antitoxin	bi	A2U21_06310	99
	A2U20_05390	*higB4*	mRNA interferase toxin HigB	bi	A2U21_06305	100
	A2U20_05455	*higB5*	mRNA interferase toxin HigB	bi	A2U21_06250	99
	A2U20_05460	*higA7*	Antitoxin HigA_mRNA interferase antitoxin	bi	A2U21_06245	100
	A2U20_05480	*cdiA*	Contact-dependent growth inhibition (CDI) toxin	-		
	A2U20_05555	*chpS*	Antitoxin ChpS	bi	A2U21_06185	99
	A2U20_05585	*tabA*	Toxin-antitoxin biofilm protein TabA	bi	A2U21_06150	100
	A2U20_05690	*hicA3*	Addiction module toxin HicA	bi	A2U21_04705	62
	A2U20_06620	*higA8*	Antitoxin HigA_mRNA interferase antitoxin	bi	A2U21_05330	93
	A2U20_06625	*higB6*	mRNA interferase toxin HigB	bi	A2U21_05325	99
	A2U20_06830	*cdiA2*	Contact-dependent growth inhibition (CDI) toxin	-		
	A2U20_06850	*cdiA3*	Contact-dependent growth inhibition (CDI) toxin	-		
	A2U20_06865	*cdiA4*	Contact-dependent growth inhibition (CDI) toxin	-		
	A2U20_06875	*cdiA5*	Contact-dependent growth inhibition (CDI) toxin	-		
	A2U20_07065	*pezT2*	Antitoxin/toxin system zeta toxin	bi	A2U21_03130	75
	A2U20_07375	*ltxB*	Leukotoxin export ATP-binding protein LtxB	uni	A2U21_07020	52
	A2U20_08285	*cdiA6*	Contact-dependent growth inhibition (CDI) toxin	-		
	A2U20_08295	*cdiA7*	Contact-dependent growth inhibition (CDI) toxin	-		
	A2U20_08515	*pasI*	Persistence and stress-resistance antitoxin PasI	bi	A2U21_04275	98
	A2U20_10085	*hicB*	Antitoxin HicB	bi	A2U21_00970	100
	A2U20_10110	*higA9*	Antitoxin HigA_mRNA interferase antitoxin	bi	A2U21_00995	98
	A2U20_10115	*higB7*	mRNA interferase toxin HigB	bi	A2U21_01000	99
	A2U20_10565	*hipA2*	Serine/threonine-protein kinase toxin HipA	bi	A2U21_00565	99
Other	A2U20_00515	*nanH*	Sialidase	bi	A2U21_11540	99
	A2U20_01700	*espP*	Putative extracellular serine protease	bi	A2U21_09655	75
	A2U20_01715	*espP2*	Putative extracellular serine protease	bi	A2U21_09640	78
	A2U20_03355	*vacJ*	Putative VacJ lipoprotein	bi	A2U21_08285	97
	A2U20_05150	*sirA*	Regulator of disulfide bond formation	bi	A2U21_06540	99
	A2U20_05155	*sirB*	Invasion protein expression up-regulator SirB	bi	A2U21_06535	100
	A2U20_07890	*sodA*	Superoxide dismutase	bi	A2U21_03770	100
	A2U20_08325	*sodC*	Superoxide dismutase	bi	A2U21_04050	92

^a^ The Hit column contains a '-' (no hit), 'uni' or 'bi' RAST server results from a one-to-one BLASTP comparison of the protein coding sequences in the Nagasaki genome using the D74 genome as the reference. “bi” represents a bidirectional best hit in which the reverse hit from the Nagasaki comparison genome to the D74 reference genome was also the best hit. “uni” indicates a uni-directional hit in which the reverse hit from the comparison genome to the reference genome was not also the best hit. “-”indicates no hit or match was found.

^b^Global pairwise nucleotide percent sequence identity.

**Table 4:** Predicted virulence-associated genes identified in *H*. *parasuis* Nagasaki.

Group	Nagasaki locus_tag	Nagasaki Name	Product/ Function	Hit^a^	D74 locus_tag	% Identity^b^
Adhesin	A2U21_02720	*aidA*	Type V secretory pathway, adhesin AidA	-		
	A2U21_03995	*fimD*	Putative F17-like fimbrial usher	bi	A2U20_08925	99
	A2U21_04065	*aidA2*	Type V secretory pathway, adhesin AidA	bi	A2U20_08340	72
	A2U21_06820	*ompA*	Outer membrane protein A precursor	bi	A2U20_04855	98
	A2U21_07040	*ompD15*	Surface antigen (D15), outer membrane protein	bi	A2U20_04655	99
	A2U21_07315	*ompP2*	Outer membrane protein P2 precursor	bi	A2U20_04380	85
	A2U21_07950	*ompP1*	Outer membrane protein precursor P1	bi	A2U20_03710	90
	A2U21_08150	*ompP5*	putative outer membrane protein P5	bi	A2U20_03490	92
	A2U21_08195	*pilQ*	Type IV pilus biogenesis protein PilQ	bi	A2U20_03445	94
	A2U21_08210	*pilN*	Type IV pilus biogenesis protein PilN	bi	A2U20_03430	98
	A2U21_08215	*pilM*	Type IV pilus biogenesis protein PilM	bi	A2U20_03425	98
	A2U21_08410	*pulJ*	Type II secretory pathway, component PulJ	bi	A2U20_03220	96
	A2U21_08415	*pulG*	Type II secretory pathway, pseudopilin PulG	bi	A2U20_03215	97
	A2U21_08925	*pilW*	Type IV pilus biogenesis/stability protein PilW	bi	A2U20_02695	100
	A2U21_10845	*pilD*	Tfp pilus assembly pathway, fimbrial leader peptidase	bi	A2U20_07225	94
	A2U21_10850	*pilC*	Type IV fimbrial assembly protein PilC	bi	A2U20_07220	99
	A2U21_10855	*pilB*	Type IV fimbrial assembly ATPase PilB	bi	A2U20_07215	98
	A2U21_10860	*pilA*	Type IV pilin PilA	bi	A2U20_07210	87
Hemolysin	A2U21_00465		Putative hemagglutinin/hemolysin-related protein	-		
	A2U21_00470	*prtB*	Serralysin B, hemolysin-type calcium-binding region	bi	A2U20_10970	69
	A2U21_02620	*ahpA*	Hemolysin regulation protein AhpA	bi	A2U20_09890	99
	A2U21_04100	*osmY*	Osmotically-inducible protein OsmY_putative hemolysin	bi	A2U20_08375	100
	A2U21_11130	*shlB1*	Hemolysin transporter protein ShlB	-		
	A2U21_11135	*shlB2*	Hemolysin transporter protein ShlB	bi	A2U20_08300	53
	A2U21_11140		putative hemolysin	-		
	A2U21_11145		Putative hemolysin	-		
	A2U21_11150	*hpmA*	Hemolysin	-		
*Secretion*	A2U21_02375		Periplasmic/secreted protein	bi	A2U20_09675	93
	A2U21_08050	*esiB*	Putative secretory immunoglobulin A-binding protein	-		
	A2U21_08055	*esiB2*	Putative secretory immunoglobulin A-binding protein	bi	A2U20_01865	51
Toxin	A2U21_00565	*hipA*	Serine/threonine-protein kinase toxin HipA	bi	A2U20_10565	99
	A2U21_00620	*ebgC*	EbgC protein_Toxin-antitoxin biofilm protein TabA	bi	A2U20_00255	98
	A2U21_00970	*hicB*	Antitoxin HicB	bi	A2U20_10085	100
	A2U21_00995	*higA*	Antitoxin HigA_mRNA interferase antitoxin	bi	A2U20_10110	98
	A2U21_01000	*higB*	mRNA interferase toxin HigB	bi	A2U20_10115	99
	A2U21_02950	*hicA*	Addiction module toxin HicA	bi	A2U20_03635	94
	A2U21_03035	*relE*	Addiction module toxin RelE	bi	A2U20_02840	59
	A2U21_03130	*pezT*	Antitoxin/toxin system zeta toxin	bi	A2U20_07065	75
	A2U21_03940		Toxin-antitoxin system, antitoxin component	-		
	A2U21_04275	*pasI*	Persistence and stress-resistance antitoxin PasI	bi	A2U20_08515	98
	A2U21_04650	*higA2*	Antitoxin HigA_mRNA interferase antitoxin	uni	A2U20_01495	54
	A2U21_04705	*hicA2*	Addiction module toxin HicA	bi	A2U20_05690	62
	A2U21_04735	*hicA3*	Addiction module toxin HicA	uni	A2U20_03635	94
	A2U21_04850	*relE*	mRNA interferase toxin RelE	-		
	A2U21_05325	*higB2*	mRNA interferase toxin HigB	bi	A2U20_06625	99
	A2U21_05330	*higA3*	Antitoxin HigA_mRNA interferase antitoxin	bi	A2U20_06620	93
	A2U21_05725	*relE2*	Addiction module toxin RelE	-		
	A2U21_05930	*hicB2*	Antitoxin HicB	-		
	A2U21_06130	*higA4*	Antitoxin HigA_mRNA interferase antitoxin	bi	A2U20_05030	98
	A2U21_06135	*higB3*	mRNA interferase toxin HigB	bi	A2U20_05035	99
	A2U21_06150	*tabA*	Putative Toxin-antitoxin biofilm protein TabA	bi	A2U20_05585	100
	A2U21_06185	*chpS*	Antitoxin	bi	A2U20_05555	99
	A2U21_06190	*pemK*	Programmed cell death toxin PemK	bi	A2U20_05550	97
	A2U21_06245	*higA5*	Antitoxin HigA_mRNA interferase antitoxin	bi	A2U20_05460	100
	A2U21_06250	*higB4*	mRNA interferase toxin HigB	bi	A2U20_05455	99
	A2U21_06305	*higB5*	mRNA interferase toxin HigB	bi	A2U20_05390	100
	A2U21_06310	*higA6*	Antitoxin HigA_mRNA interferase antitoxin	bi	A2U20_05385	99
	A2U21_06560	*cdtA*	Toxin	bi	A2U20_04835	99
	A2U21_06565	*cdtB*	Cytolethal distending toxin subunit CdtB	bi	A2U20_05125	96
	A2U21_06570	*cdtC*	Toxin	bi	A2U20_04825	99
	A2U21_06840	*cdtA2*	Toxin	uni	A2U20_04835	99
	A2U21_06845	*cdtB2*	Cytolethal distending toxin subunit CdtB	uni	A2U20_04830	94
	A2U21_06850	*cdtC2*	Toxin	uni	A2U20_04825	94
	A2U21_08015	*relE3*	Addiction module toxin RelE	-		
	A2U21_08020	*stbD*	RelB/StbD replicon stabilization protein; antitoxin to RelE/StbE	-		
	A2U21_08785	*tdeA*	Putative memebrane toxin/ drug export protein A	bi	A2U20_02805	97
	A2U21_08910	*higB6*	mRNA interferase toxin HigB	bi	A2U20_02710	96
	A2U21_08915	*higA7*	Antitoxin HigA_mRNA interferase antitoxin	bi	A2U20_02705	93
	A2U21_09500	*hipA2*	Toxin HipA	bi	A2U20_02115	95
	A2U21_09900	*vapD*	Virulence-associated protein D; endoribonuclease	-		
	A2U21_10000	*vapC*	VapC toxin family PIN domain ribonuclease	bi	A2U20_01850	78
Other	A2U21_03770	*sodA*	Superoxide dismutase	bi	A2U20_07890	100
	A2U21_04050	*sodC*	Superoxide dismutase	bi	A2U20_08325	92
	A2U21_06535	*sirB*	Invasion protein expression up-regulator SirB	bi	A2U20_05155	100
	A2U21_06540	*sirA*	Regulator of disulfide bond formation	bi	A2U20_05150	99
	A2U21_08285	*vacJ*	putative VacJ lipoprotein	bi	A2U20_03355	97
	A2U21_09640	*espP*	Putative serine protease	bi	A2U20_01715	78
	A2U21_09655	*espP2*	Putative serine protease	bi	A2U20_01700	75
	A2U21_11540	*nanH*	Sialidase	bi	A2U20_00515	99

^a^ The Hit column contains a '-' (no hit), 'uni' or 'bi' RAST server results from a one-to-one BLASTP comparison of the protein coding sequences in the Nagasaki genome using the D74 genome as the reference. “bi” represents a bidirectional best hit in which the reverse hit from the Nagasaki comparison genome to the D74 reference genome was also the best hit. “uni” indicates a uni-directional hit in which the reverse hit from the comparison genome to the reference genome was not also the best hit. “-”indicates no hit or match was found.

^b^Global pairwise nucleotide percent sequence identity.

Eight CDSs encoding predicted hemolysins were identified in D74 including two (locus tags A2U20_06885 and A2U20_07380) unique to strain D74 (Table 3). D74 harbors two putative Serralysin B genes, *prtB* and *prtB2*, and a putative Serralysin C gene *prtC*. In contrast, only putative Serralysin B gene *prtB* was identified in strain Nagasaki (Table 4). A notable size difference appears to exist between the proteins encoded by both *prtB*, *prtB2*, *and prtC* in D74, 1,954, 1,703, and 1,911 amino acids respectively, compared to the putative Serralysin B 910 amino acid protein encoded by *prtB* in Nagasaki. Nine CDSs encoding predicted hemolysins were identified in Nagasaki, five of which were identified as unique to Nagasaki. These include *hpmA* (A2U21_11150) and locus-tags A2U21_00465, A2U21_11140, and A2U210_11145. Nagasaki harbors two putative hemolysin transporter genes *shlB1* and *shlB2* (chromosomally located next to each other), while D74 contains only *shlB1* gene (Tables 3 and 4). A notable size difference was also observed for the *shlB* gene in D74 encoding a putative 582 amino acid protein compared to the putative 329 amino acid protein encoded by the *shlB* in Nagasaki.

Two CDSs encoding genes predicted to function in secretion were identified in D74, A2U20_09675 and *esiB*, a putative secretory immunoglobulin A-binding encoding gene, while three were identified in Nagasaki. These include A2U21_02375 and two putative secretory immunoglobulin A-binding encoding genes *esiB* and *esiB2*, chromosomally located next to each other.

Thirty-nine CDSs encoding predicted toxins were identified in D74 and forty-six CDSs encoding predicted toxins were identified in Nagasaki. A noticeable difference between the CDSs encoding predicted toxins identified in D74 and Nagasaki was the seven *cdiA* genes encoding putative contact-dependent growth inhibition toxin A harbored only in strain D74 (Table 3). These *cdiA* genes harbored by D74 are discussed in more detail below.

Both strains harbored a large number of toxin-antitoxin (TA) systems. TA systems are small genetic elements comprised of two components, a stable protein toxin and its more labile antagonistic antitoxin, which can be a protein or non-coding RNA [52]. TA systems were originally identified as plasmid-borne loci, which functioned to promote plasmid maintenance by killing daughter cells that lacked the TA encoded plasmid [52]. TA loci were subsequently discovered in numerous bacterial and archaeal chromosomes and provide several functions, such as stabilization of genomic regions, anti-addiction against similar plasmid-borne toxins, defense against phage infection, biofilm formation, control of the stress response, and bacterial persistence [52–54]. Six types of TA systems (types I to VI) have been described to date based on the type (either RNA or protein) and mode of action of the antitoxin [53]. The Type II TA system is highly abundant among prokaryotes and has been extensively studied [52, 54–56]. In type II TA systems, both toxin and antitoxin are small proteins encoded by genes in a bicistronic operon [52, 54–56]. The antitoxin blocks the toxicity of the toxin by forming a complex with it [52, 54–56]. Both D74 and Nagasaki contain several Type II TA families including *relBE*, *mazEF*, *vapBC*, and *higBA*, which was the most abundant with 10 putative *higBA* loci identified in D74 and 6 putative *higBA* loci identified in Nagasaki, with a varying degree of similarity (Table 3 and Table 4).

Eight orthologs of other virulence-associated proteins were identified in both strains (Table 3 and Table 4). A 75% sequence identity was observed between corresponding orthologs *espP* (A2U20_01700) in D74 and *espP2* (A2U21_09655) in Nagasaki and a 78% sequence identity was observed between corresponding orthologs *espP2* (A2U20_01715) in D74 and *espP* (A2U21_09640) in Nagasaki (Table 3 and Table 4). Additionally a notable size difference was also observed for the putative proteins encoded by *espP* (A2U20_01700), 1,071 amino acids, and *espP2* (A2U20_01715), 985 amino acids, in D74, compared to the putative proteins encoded by *espP* (A2U21_09640), 781 amino acids, and *espP2* (A2U21_09655), 772 amino acids, in Nagasaki.

### *cdiA* genes encoding putative contact-dependent growth inhibition proteins identified in *H*. *parasuis* D74

Contact-dependent growth inhibition (CDI) is a process used by Gram-negative bacteria to deliver diverse growth inhibiting nuclease toxins into the cytoplasm of neighboring cells upon cell-cell contact [57–61]. CDI is mediated by the CdiB and CdiA proteins, which are members of the TpsB and TpsA two-partner secretion (TPS) group of proteins [61–63]. CdiB facilitates secretion of the CdiA “exoprotein” onto the cell surface [61, 64]. CdiA then binds to specific outer-membrane receptors on susceptible bacteria and transfers its C-terminal toxin domain (CdiA-CT) into the target cell [58–61]. CDI+ bacteria also produce small immunity proteins (CdiI) that protect them from toxin delivered by neighboring cells of closely related species or sibling cells by binding to the CdiA-CT and neutralizing its toxin activity [58–61].

**Fig 3:**
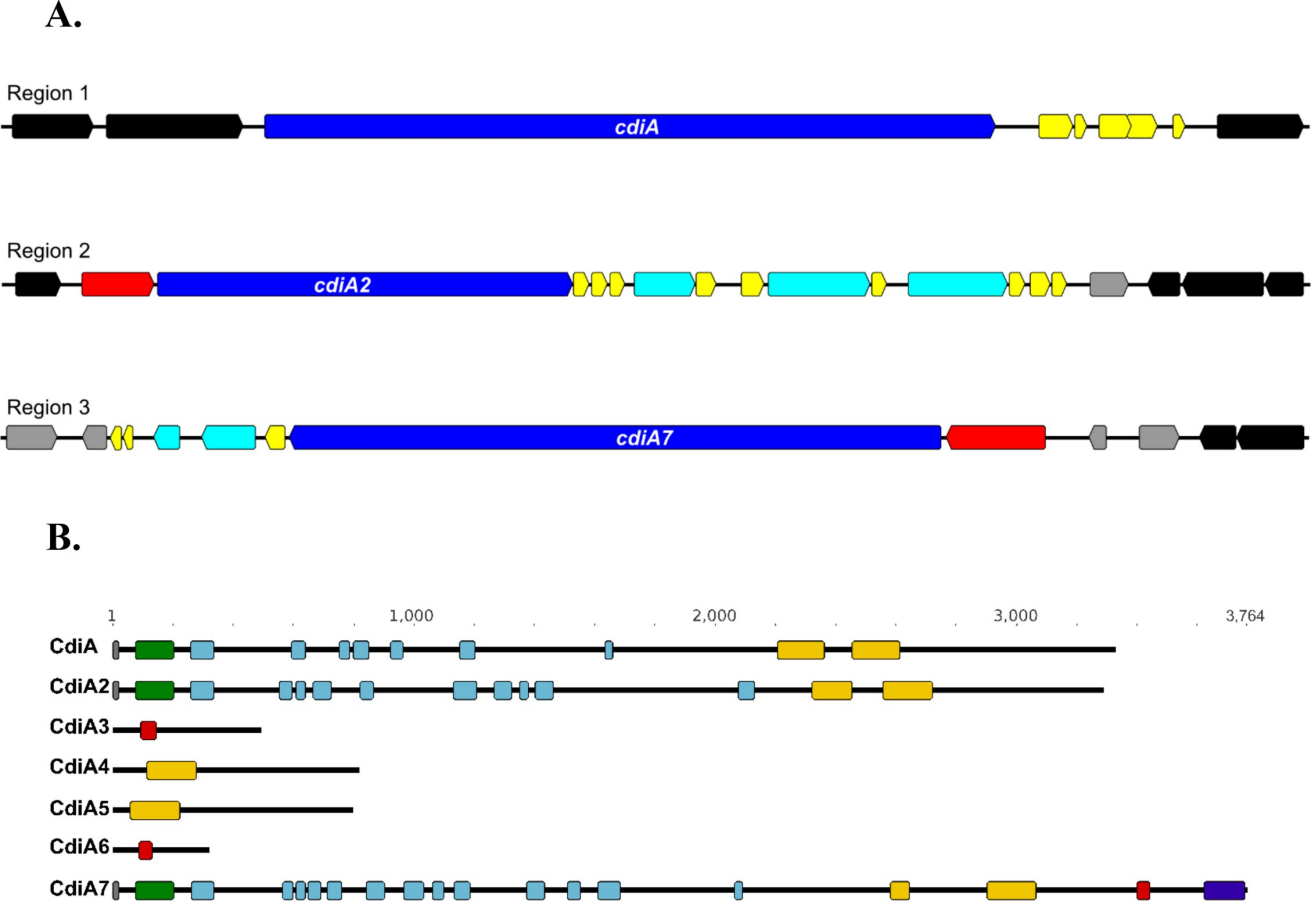
*cdiA* genes encoding putative contact-dependent growth inhibition proteins identified in *H*. *parasuis* D74. (A) Organization of *cdi* loci in D74. The three genomic regions containing putative *cdiA* genes are depicted. Gene function was assigned based on the results of BLASTX searches. Each arrow represents a gene within the locus; direction of the arrow indicates orientation within the closed genome sequence. Dark blue arrows represent the *cdiA* genes, red arrows represent *cdiB* homologues, light blue arrows represent potential “orphan” *cdiA* genes, yellow arrows represent putative *cdiI* candidates, and grey arrows represent genes predicted to have functions relating to horizontal gene transmission, black arrows represent genes whose predicted function is unrelated to contact-dependent inhibition. Genomic regions are not shown to scale. (B) Domain architecture of the predicted CdiA proteins. Domain content of the CdiA proteins was determined using a pfam database search. Grey boxes represent ESPR signal peptide domains (PF13018), green boxes represent Haemagg_act domains (PF05860), light blue boxes represent Fil_haemagg domains (PF05594), orange boxes represent Fil_haemagg_2 domains (PF13332), red boxes represent PT-VENN domains (PF04829), and purple boxes represent EndoU_bacteria domains (PF14436).

The putative *cdiA* genes in strain D74 are located in three regions with a *cdiBAI* organization with “orphan” *cdiA* genes located downstream, which is similar to the organization found in *E*. *coli* strains and other gamma-proteobacteria [61, 65]. Region one includes *cdiA* (A2U20_05480) and five upstream potential *cdiI* candidate genes (locus tags A2U20_05485-A2U20_05505) (Fig 3A). The second region encompasses *fhaC* (A2U20_06825) located upstream of *cdiA2* (A2U20_06830), which, consistent with other TpsB proteins, shares substantial homology with CdiB (Fig 3A) [66]. Nine potential *cdiI* candidate genes (locus tags A2U20_06835-A2U20_06845, A2U20_06855-A2U20_06860, A2U20_06870, and A2U20_06880-A2U20_06890) and *cdiA3*, *cdiA4*, and *cdiA5* (locus tags A2U20_06850, A2U20_06865, A2U20_06875) are located downstream of *cdiA2*. Genes *cdiA3*, *cdiA4*, and *cdiA5* were identified as “orphan” *cdiA* genes based on their shared characteristics such as smaller size compared to larger full-length *cdiA* genes and resemble the 3’-ends of larger *cdiA* genes. (Fig 3A) [65]. The third region contains the *cdiA7* (A2U20_08295) gene with the *shlB* gene (A2U20_08300) gene located upstream (Fig 3). Similar to FhaC and other TpsB proteins, ShlB shares substantial homology with CdiB [66]. A potential *cdiI* candidate (A2U20_08290), followed by two potential “orphan” *cdiA* genes, *cdiA6* (A2U20_08285) and a predicted pseudogene *fha* (A2U20_08280), followed by two potential *cdiI* candidate genes (locus tags A2U20_08270 and A2U20_08265) are located downstream of *cdiA7* (Fig 3A).

CdiA proteins share a number of characteristics in common with the TpsA family protein FHA [61, 67]. CdiA proteins typically contain an N-terminal region homologous to FHA containing the TPS domain required for interaction with the TpsB partner, CdiB or FhaC respectively, and haemagglutinin repeats that are predicted to form a β-helical structure [61, 67]. In addition, most *cdiA* homologues encode the VENN peptide motif, which delineates the beginning of C-terminal toxin domain as well as the conserved and variable regions [61, 67]. The predicted proteins encoded by the *cdiA* genes in D74 were evaluated for the presence of these domains. Domains identified in CdiA include a ESPR signal peptide domain (PF13018) required for export and multiple haemaglutination activity domains, specifically one Haemagg_act domain (PF05860), seven Fil_haemagg domains (PF05594), two Fil_haemagg_2 domains (PF13332) (Fig 3B). CdiA2 is similar to CdiA and contains a ESPR signal peptide domain (PF13018) required for export and multiple haemaglutination activity domains, including one Haemagg_act domain (PF05860), ten Fil_haemagg domains (PF05594), two Fil_haemagg_2 domains (PF13332) (Fig 3B). CdiA3 and CdiA6 are similar to each other and contain a pre-toxin domain with VENN motif that marks the beginning of the C-terminal toxin domain similar to other previously reported CdiA proteins (Fig 3B). CdiA4 and CdiA5 are also similar to each other and both contain a Fil_haemagg_2 domain (PF13332) (Fig 3B). CdiA7 is the largest CdiA protein in D74 and contains a ESPR signal peptide domain (PF13018) required for export and multiple haemaglutination activity domains, including one Haemagg_act domain (PF05860), thirteen Fil_haemagg domains (PF05594), two Fil_haemagg_2 domains (PF13332), a pre-toxin VENN domain (PF04829), and a EndoU_bacterial nuclease domain (PF14436) (Fig 3B). Overall, *cdiA3*, *cdiA4*, *cdiA5*, and *cdiA6* are similar to previously reported "orphan" *cdiA* genes as they encode much smaller proteins that lack a conserved export signal and other functional domains. The region containing the orphan *cdiA* genes was further evaluated and no additional cdi-related protein domains were found beyond those depicted in Fig 3B for CdiA3, CdiA4, CdiA5, and CdiA6. This indicates that the orphan *cdiA* genes are not the result of frameshift or indel mutations causing disruption of a larger intact *cdiA* gene(s). This genomic organization of a *cdi* locus containing one or more orphan *cdiA* genes downstream of full-length *cdiA* is common in many species of bacteria [65]. In contrast, CdiA, CdiA2, and CdiA7 are larger and contain most of the functional domains harbored by previously characterized CdiA-CT proteins; however, CdiA7 shares more similarity to other CdiA-CT proteins in that it additionally contains a PT-VENN motif and a recognizable nuclease domain at C-terminus.

### *vtaA* family of trimeric autotransporter genes identified in *H*. *parasuis* strains D74 and Nagasaki

Pina et al. first identified thirteen proteins encoded by the *vtaA* family of trimeric autotransporter genes in strain Nagasaki based on the occurrence of a C-terminal YadA anchor domain, which defines this family of proteins [15]. That study also identified 17 homologues harbored by other *H*. *parasuis* strains with relatively conserved sequence within the passenger domain among *vtaA* homologues from pathogenic isolates and a high degree of divergence among non-virulent isolates [15]. The authors subsequently named these genes *vtaA* or virulence-associated trimeric autotransporter genes and classified the proteins encoded by *vtaA* genes into three groups based on sequence comparison of the C-terminal YadA anchor domain, with groups 1 and 2 being strongly associated with virulent *H*. *parasuis* isolates [15]. Previous studies have demonstrated that VtaA proteins are involved in virulence as well as being immunogenic, are produced during an infection, and are capable of conferring protection [68–70]. Recently, a comparison between the D74 and Nagasaki draft genomes identified only three *vtaA* genes harbored by D74 compared to thirteen harbored by Nagasaki [34]. Unfortunately, many *vtaA* genes identified within each strain were incomplete in the draft sequences, preventing a reliable one-to-one assignment of the *vtaA*-like ORFs to specific *vtaA* genes and subsequent evaluation of the predicted protein structure.

**Fig 4:**
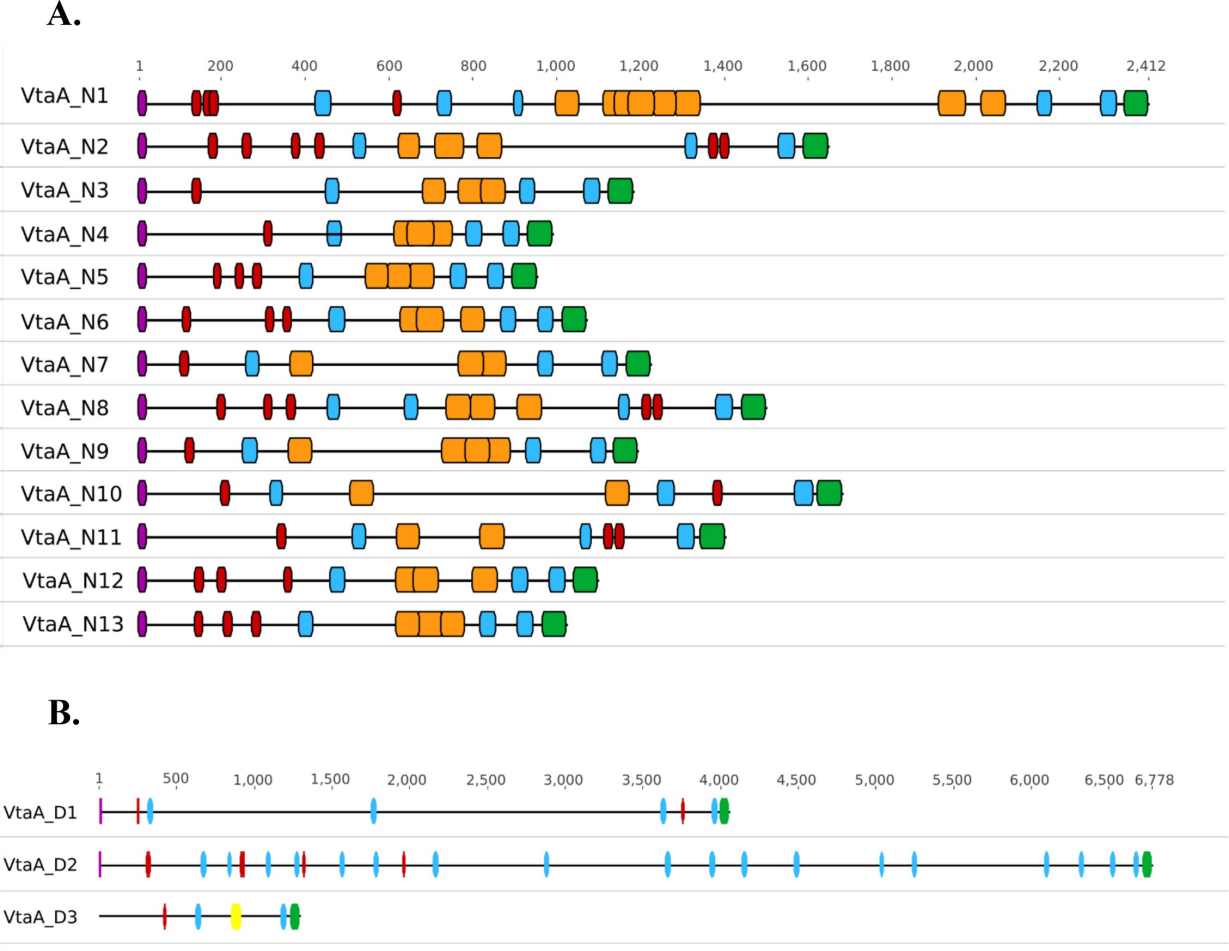
Domain architecture of the predicted VtaA proteins in *H*. *parasuis* strains D74 and Nagasaki. (A) Domain architecture of the predicted VtaA proteins from Nagasaki. Assigned gene name designations are shown at left. Schematic depictions of the 13 Nagasaki VtaA proteins is shown. The domains were identified by a pfam database search. ESPR signal peptides (PF13018) are shown in purple, YadA head domains (PF05658) in red, YadA stalk domains (PF05662) in blue, YadA anchor domains (PF03895) in green, collagen domains (PF1391) in orange, TAA-Trp-ring domains (PF15401) in yellow. (B) Domain architecture of the predicted VtaA proteins from D74. Assigned gene name designations are shown at left. Schematic depictions of the pfam domains, colored as in Panel A, for the 3 VtaA proteins from D74.

To ensure identification of all potential *vtaA* genes, all protein coding sequences in Nagasaki and D74 were searched for the occurrence of a YadA anchor domain and no additional YadA anchor domain containing proteins were identified. The 13 *vtaA* genes identified in Nagasaki have been named according to their location along the chromosome and are listed in Table 5 along with their respective name and group originally assigned by Pina et al. [15]. Similarly, the 3 *vtaA* genes identified in D74 have been named according to their location along the chromosome and are also listed in Table 5. The predicted protein structure of all VtaA proteins for both Nagasaki and D74 were evaluated for known domain content (Fig 4). All Nagasaki VtaA proteins contain an N-terminal extended signal peptide or ESPR domain (PF13018) for Type V secretion, followed by 1–4 YadA head domains (PF05658), and 3–5 YadA anchor domains (PF03895). This region containing the head and stalk domains is referred to as the passenger domain region [71]. Following the passenger domain region, all Nagasaki VtaA proteins contain 2–8 collagen triple helix repeat domains (PF01391) followed by the C-terminal YadA anchor domain (PF03895) (Fig 4A). In contrast, none of the D74 VtaA proteins contain a collagen triple helix repeat domain (PF01391) and the predicted size for both VtaA_D1 (4054 AA) and VtaA_D2 (6778 AA) proteins is substantially larger than any predicted Nagasaki VtaA protein (Fig 4B). Additionally, VtaA_D3 differs from VtaA_D1 and VtaA_D2 proteins. VtaA_D3 contains a tryptophan-ring motif domain or TAA-Trp-ring (PF15401) and does not contain an N-terminal ESPR domain (PF13018) (Fig 4B). The absence of the ESPR domain suggests that VtaA_D3 may not be exported across the inner membrane. When we compared our VtaA predicted protein sequences to those reported by Pina et al. [15] two differences were identified and the other 11 out of 13 sequences were found to be 100% identical. The two differences identified were an amino acid change in VtaA_N9 (S1191G) relative to the previously reported sequence and an 18 amino acid insertion (AGPTGPQGPAGPTGSQDP) after amino acid 737 of VtaA8 that is not present in our VtaA_N4 sequence. The absence or presence of the insertion is located within the third collagen repeat domain and does not affect the presence or absence of this domain nor the total number of collagen repeat domains predicted for the two proteins.

**Table 5:** *vtaA* genes identified in *H*. *parasuis* Nagasaki.

Nagasaki locus_tag	Nagasaki Name	Name assigned by Pina et al. [15]	Group assigned by Pina et al. [15]	Hit^a^	D74 locus_tag	D74 Name	% Identity^b^
A2U21_00055	vtaA_N1	vtaA1	1	-			0
A2U21_00360	vtaA_N2	vtaA10	2	uni	A2U20_04585	vtaA_D1	49.73
A2U21_00905	vtaA_N3	vtaA4	1	-			0
A2U21_03315	vtaA_N4	vtaA8^**c**^	1	bi	A2U20_05995	vtaA_D3	36.84
A2U21_04400	vtaA_N5	vtaA9	1	uni	A2U20_04585	vtaA_D1	30.74
A2U21_05305	vtaA_N6	vtaA6	1	-			0
A2U21_05400	vtaA_N7	vtaA2	1	-			0
A2U21_05640	vtaA_N8	vtaA11	2	uni	A2U20_04585	vtaA_D1	50.94
A2U21_06175	vtaA_N9	vtaA3^**d**^	1	-			0
A2U21_06350	vtaA_N10	vtaA12	1	bi	A2U20_04585	vtaA_D1	94.09
A2U21_07110	vtaA_N11	vtaA13	1	uni	A2U20_04585	vtaA_D1	59.87
A2U21_08125	vtaA_N12	vtaA5	3	-			0
A2U21_09355	vtaA_N13	vtaA7	3	-			0

^a^ The Hit column contains a '-' (no hit), 'uni' or 'bi' RAST server results from a one-to-one BLASTP comparison of the protein coding sequences in the D74 genome using the Nagasaki genome as the reference. “bi” represents a bidirectional best hit in which the reverse hit from the D74 comparison genome to the Nagasaki reference genome was also the best hit. “uni” represents uni-directional indicates a hit in which the reverse hit from the comparison genome to the reference genome was not also the best hit. “-”indicates no hit or match was found.

^b^Local percent sequence identity.

^c^18 amino acid insertion compared to the Pina et al. [15] sequence.

^d^ Single amino acid difference at position 1191.

When the genome regions in Nagasaki and D74 were compared, the sequence and gene content up and downstream of the *vtaA* gene locations in Nagasaki were similar to the reciprocal locations in D74. No evidence of extensive genome rearrangement at these locations was observed. A different gene or genes were identified in D74 at the same location of a reciprocal *vtaA* gene in Nagasaki, which could have arisen from small insertion events. Further evaluation of the *vtaA* genes in both strains suggested that they are not in an operon configuration. Pina et al. identified an active promoter upstream of *vtaA*_N3 [15]. *In silico* analysis indicated that the upstream region of all 13 Nagasaki *vtaA* genes contained highly similar promoter sequences to the *vtaA*_N3 promoter. A highly similar promoter sequence to the *vtaA*_N3 was additionally observed upstream of *vtaA*_D2 in D74. This sequence conservation implies similar expression and/or regulation mechanisms among these genes.

### Methylation motifs and RM-systems in *H*. *parasuis* strains D74 and Nagasaki

In bacteria, the most common post-replicative modification of DNA is methylation by methyltransferase (MTase) enzymes resulting in three types of epigenetic markers: N6-methyladenine (m6A), N4-methylcytosine (m4C) and 5-methylcytosine (m5C) [47, 72]. DNA methylation serves several key roles in bacterial processes, including mismatch repair, the timing of DNA replication, conferring protection against bacteriophages, and regulating gene expression. [73–78]. Analysis of the SMRT DNA sequencing kinetics was used to identify total base modifications in the genomes of *H*. *parasuis* D74 and Nagasaki, and the modified sequence motifs for each strain are summarized in Tables 6 and 7.

**Table 6:** Methylation motifs detected in *H*. *parasuis* D74.

Motif^a^	Modification Type	# Detected	# in Genome	% Detected	Mean Modification QV	Mean Motif Coverage	Partner Motif
**A**GCNNNNNGCT	m6A	767	772	99.4%	535.6	376.2	AGCNNNNNGCT
GT**A**NNNNNNTGG	m6A	817	825	99.0%	436.6	380.3	CCANNNNNNTAC
CC**A**NNNNNNTAC	m6A	813	825	98.5%	421.3	381.9	GTANNNNNNTGG
**A**AGCTT	m6A	623	630	98.9%	442.4	384.0	AAGCTT
G**A**TC	m6A	15,534	15,718	98.8%	410.7	381.5	GATC
GT**A**HNNNNNNCTTG	m6A	217	220	98.6%	420.1	378.9	
CA**A**GNNNNNGNTAC	m6A	55	57	96.5%	397.1	348.8	
**A**GCNNNNGGATC	m6A	47	55	85.5%	346.8	365.8	
GC**A**GGVNNDG	m6A	316	659	48.0%	99.1	382.2	
V**A**AGCTCKD	m6A	207	447	46.3%	131.9	391.1	
**A**HBYAGYAD	m6A	662	2,632	25.2%	108.6	376.7	
DD**T**GTNDNDG	modified_base^b^	1,575	8,346	18.9%	52.3	356.8	
**T**NNNNNNH	modified_base^b^	179,073	1,197,696	15.0%	52.7	367.5	
D**T**NVVNDDG	modified_base^b^	9,920	78,834	12.6%	48.6	366.4	
**A**GNNNNNH	m6A	15,854	200,853	7.9%	112.6	371.8	

^a^Bold underlined bases indicate methylated base in motif sequence.

^b^Base modification not identified or recognized by software.

**Table 7:** Methylation motifs detected in *H*. *parasuis* Nagasaki.

Motif^a^	Modification Type	# Detected	# in Genome	% Detected	Mean Modification QV	Mean Motif Coverage	Partner Motif
A**A**GNNNNNCTT	m6A	1,322	1,324	99.8%	719.7	571.6	AAGNNNNNCTT
A**A**CNNNNNTGG	m6A	1,089	1,092	99.7%	676.2	592.7	CCANNNNNGTT
CC**A**NNNNNGTT	m6A	1,088	1,092	99.6%	700.0	622.7	AACNNNNNTGG
GT**A**NNNNNNNCTTG	m6A	217	218	99.5%	641.8	610.1	CAAGNNNNNNNTAC
CA**A**GNNNNNNNTAC	m6A	215	218	98.6%	671.3	582.9	GTANNNNNNNCTTG
G**A**TC	m6A	14,603	14,778	98.8%	602.6	617.8	GATC
**A**GGNNNNNCCT	m6A	454	460	98.7%	688.5	590.4	AGGNNNNNCCT
**A**AGVNNNNCTT	m6A	303	1,013	29.9%	130.6	608.1	
R**A**HDBAGYA	m6A	721	2,881	25.0%	107.0	582.3	
**T**NNNNNNH	modified_base^b^	181,179	1,123,132	16.1%	58.7	579.9	
DD**T**NVVNDDG	modified_base^b^	9,439	61,834	15.3%	54.8	582.8	
**T**SNNKNNG	modified_base^b^	7,871	64,478	12.2%	52.9	587.1	
**A**GDNNNNH	m6A	11,510	134,477	8.6%	178.5	591.9	
B**A**GCNVNNH	m6A	1,133	24,217	4.7%	140.2	629.3	

^a^Bold underlined bases indicate methylated base in motif sequence.

^b^Base modification not identified or recognized by software.

A total of 15 sequence motifs were identified in strain D74, and N6-methyladenine (m6A) was the most prevalent type of modification detected (Table 6). Focusing on strain Nagasaki, a total of 14 recognition sites for methylation or sequence motifs were identified and, similar to D74, N6-methyladenine (m6A) was the most prevalent type of modification detected (Table 7). This analysis revealed a surprising degree of diversity in motifs observed between these closely related strains given that the methylation motif 5’-GA^m6^TC-3’ was the only motif shared or observed in both D74 and Nagasaki (Table 6 and Table 7).

The genomes of *H*. *parasuis* Nagasaki and D74 were assessed using the Restriction Enzyme Database REBASE (www.rebase.neb.com) [47] for determination of putative MTases involved with each motif and for comparisons with known modification systems. A total of 26 genes associated with restriction-modification systems were identified in *H*. *parasuis* D74, including 11 genes associated with Type 1 restriction-modification (RM) systems and 15 genes associated with Type II RM systems (Table 8). Genes associated with Type III RM systems were not identified in D74 (Table 8). REBASE predicted three recognition sequences corresponding to a specific motif detected by the SMRT sequencing analysis. REBASE analysis indicated that the putative Type I RM enzymes S.Hpa74III, Hpa74III, and M.Hpa74III were predicted to be responsible for the 5’-GTA^m6^NNNNNNNCTTG-3’ modification (Table 8). The putative Type II RM enzymes M.Hpa74I and M.Hpa74IP were indicated by REBSE to be responsible for the motif 5’-A^m6^A GCTT-3’modification (Table 8). The putative Type II RM enzyme *dam* or Hpa74II was predicted to be responsible for the 5’-GA^m6^TC-3’modification for D74 (Table 8). The remaining two motifs detected by the SMRT sequencing analysis did not correspond with a REBASE predicted recognition sequence. Two putative Type II RM enzymes Hpa74ORFHP and M.Hpa74ORFHP were predicted by REBASE to recognize the sequence motif 5’-GGCC-3, which was not a motif detected by the SMRT sequencing analysis. Three putative RM enzyme genes identified in D74 by REBSAE analysis are predicted pseudogenes. These include S2.Hpa74ORFDP, Hpa74ORFJP, and M.Hpa74IP, associated with the Type II RM system indicated by REBSE to be responsible for the motif 5’-A^m6^A GCTT-3’modification (Table 8). In contrast, none of the putative RM enzymes genes identified in Nagasaki by REBASE analysis are predicted pseudogenes.

**Table 8:** Putative *H*. *parasuis* D74 restriction modification systems.

Type^a^	Gene^b^	D74 locus_tag	D74 Name	Predicted Recognition Sequence	REBASE Name
I	M	A2U20_05240	hsdM		M.Hpa74ORFFP
I	S	A2U20_05245	hsdS		S.Hpa74ORFFP
I	R	A2U20_05260	hsdR2		Hpa74ORFFP
I	R	A2U20_10200	hsdR3		Hpa74ORFIP
I	S	A2U20_10215	hsdS2		S1.Hpa74ORFIP
I	S	A2U20_10220	hypothetical protein CDS		S2.Hpa74ORFIP
I	M	A2U20_10230	hsdM2		M.Hpa74ORFIP
I	R	A2U20_10460^c^	hsdR4^c^		Hpa74ORFJP
I	S	A2U20_11315	hsdS3	GTANNNNNNNCTTG	S.Hpa74III
I	R	A2U20_11320	hsdR5	GTANNNNNNNCTTG	Hpa74III
I	M	A2U20_11330	hsdM3	GTANNNNNNNCTTG	M.Hpa74III
II	M	A2U20_00375	hindIIIM	AAGCTT	M.Hpa74I
II	R	A2U20_00380^c^	hindIIIR^c^	AAGCTT	Hpa74IP
II	RM	A2U20_00575	bcgIA		Hpa74ORFBP
II	S	A2U20_00580	bcgIB		S.Hpa74ORFBP
II	S	A2U20_00800	bcgIB		S1.Hpa74ORFDP
II	S	A2U20_00805^c^	bcgIB2^c^		S2.Hpa74ORFDP
II	M	A2U20_03405	dam	GATC	Hpa74II
II	RM	A2U20_07465	hypothetical protein CDS		Hpa74ORFGP
II	R	A2U20_07700	restriction endonuclease CDS	GGCC	Hpa74ORFHP
II	M	A2U20_07705	haeIIIM	GGCC	M.Hpa74ORFHP
II	M	A2U20_10010	yhdJ		M.Hpa74ORFOP
II	M	A2U20_10475	modification methylase CDS		M.Hpa74ORFLP
II	R	A2U20_10480	HNH endonuclease CDS		Hpa74ORFLP
II	S	A2U20_10485	bcgIB3		S.Hpa74ORFMP
II	RM	A2U20_10490	bcgIA2		Hpa74ORFMP

^a^Systems were designated Type I, II, or III based on REBASE analyses.

^b^Gene designations of methylase (M), restriction (R), fused restriction-modification (RM), or specificity (S), along with predicted recognition sequence and REBASE name, were determined using REBASE analysis.

^C^Predicted pseudogene.

Focusing on *H*. *parasuis* Nagasaki, 34 genes associated with restriction-modification systems were identified, including 14 genes associated with Type 1, 15 genes associated with Type II, and 5 genes associated with Type III RM systems (Table 9). Only two of the REBASE predicted recognition sequences corresponded to a specific motif detected by the SMRT sequencing analysis. REBASE analysis indicated that the putative Type I RM enzymes M.HpaNNII, HpaNNIIP, and S.HapNNII were predicted to be responsible for the 5’-GTA^m6^NNNNNNNCTTG-3’ modification (Table 9). The putative Type II RM enzyme *dam* or M.HpaNNI was predicted to be responsible for the 5’-GA^m6^TC-3’modification (Table 9). The remaining five motifs identified by the SMRT sequencing analysis represent yet unknown recognition sequences.

**Table 9:** Putative *H*. *parasuis* Nagasaki restriction modification systems.

Type^a^	Gene^b^	Nagasaki locus_tag	Nagasaki Name	Predicted Recognition Sequence^b^	REBASE Name^b^
I	M	A2U21_00095	hsdM	GTANNNNNNNCTTG	M.HpaNNII
I	R	A2U21_00110	hsdR	GTANNNNNNNCTTG	HpaNNIIP
I	S	A2U21_00115	hsdS	GTANNNNNNNCTTG	S.HpaNNII
I	R	A2U21_01085	hsdR2		HpaNNORFEP
I	S	A2U21_01100	hsdS2		S1.HpaNNORFEP
I	S	A2U21_01105	hypothetical protein CDS		S2.HpaNNORFEP
I	M	A2U21_01115	type I restriction-modification system subunit M CDS		M.HpaNNORFEP
I	R	A2U21_04210	hsdR4		HpaNNORFIP
I	S	A2U21_04220	hsdS3		S1.HpaNNORFEP
I	S	A2U21_04225	hsdS4		S2.HpaNNORFEP
I	M	A2U21_04230	hsdM2		M.HpaNNORFEP
I	R	A2U21_06440	hsdR5		HpaNNORFJP
I	S	A2U21_06445	hsdS5		S.HpaNNORFJP
I	M	A2U21_06450	hsdM3		M.HpaNNORFJP
II	S	A2U21_00855	bcgIB		S1.HpaNNORFDP
II	S	A2U21_00860	bcgIB2		S2.HpaNNORFDP
II	RM	A2U21_00865	bcgIA		HpaNNORFDP
II	M	A2U21_03345	hypothetical protein CDS		M.HpaNNORFGP
II	M	A2U21_03850	hhalM	GCGC	M.HpaNNORFHP
II	R	A2U21_03855	type II RM endonuclease	GCGC	HpaNNORFHP
II	M	A2U21_06885	hpaIIM	CCGG	M.HpaNNORFAP
II	M	A2U21_08235	dam	GATC	M.HpaNNI
II	M	A2U21_09040	bspRIM	CGCG	M.HpaNNORFLP
II	R	A2U21_09045	hypothetical protein CDS	CGCG	HpaNNORFLP
II	M	A2U21_09985	restriction endonuclease subunit M CDS		M1.HpaNNORFMP
II	M	A2U21_09990	restriction endonuclease CDS		M2.HpaNNORFMP
II	RM	A2U21_09995	restriction endonuclease CDS		HpaNNORFMP
II	M	A2U21_10160	hypothetical protein CDS		M.HpaNNORFNP
II	M	A2U21_10520	hypothetical protein CDS	BA	M.HpaNNORFOP
III	M	A2U21_01380	restriction endonuclease subunit M CDS	GGAG	M1.HpaNNORFFP
III	M	A2U21_01385	bamHIM	GGAG	M2.HpaNNORFFP
III	R	A2U21_01390	type III restriction endonuclease subunit R CDS		HpaNNORFFP
III	M	A2U21_11010	site-specific DNA-methyltransferase CDS		M.HpaNNORFPP
III	R	A2U21_11015	restriction endonuclease CDS		HpaNNORFPP

^a^Systems were designated Type I, II, or III based on REBASE analyses.

^b^Gene designations of methylase (M), restriction (R), fused restriction-modification (RM), or specificity (S), along with predicted recognition sequence and REBASE name, were determined using REBASE analysis.

Expression of MTases can undergo phase variation by slipped-strand mispairing due to the presence of simple sequence repeats (SSRs), such as homopolymeric tracts [79–83]. All of the putative RM genes identified in D74 (Table 8) and Nagasaki (Table 9) were search for the presence of SSRs within the coding region and in the region encompassing 150 bp upstream of the putative start codon. No SSRs were observed in the upstream region for five RM genes identified in D74 (A2U20_00805c, A2U20_10480, A2U20_10215, A2U20_11315, and A2U20_10200), while six homopolymeric tracts of consisting of five or more bases were observed in the upstream region of *hsdR2* (A2U20_05260) (S6 Table). SSRs were observed within the coding region of all of the D74 RM genes and the numbers of SSRs ranged from two (A2U20_00580 and A2U20_11330) to 38 (A2U20_07465) (S6 Table). No SSRs were observed in the upstream region for six RM genes identified in Nagasaki (A2U21_00115, A2U21_01085, A2U21_04210, A2U21_03345, A2U21_03850, A2U21_06885), while six homopolymeric tracts of consisting of five or more bases were observed in the upstream region of A2U21_09985 (S7 Table). SSRs were observed within the coding region of all of the Nagasaki RM genes and the numbers of SSRs ranged from two (A2U21_10520) to 31 (A2U21_04210 and A2U21_00110) (S7 Table). While further studies are warranted, the occurrence of these homopolymeric tracts within these regions indicates the potential of these genes to undergo phase variation by slip strand mispairing.

## Conclusions

This report provides the closed whole-genome sequence annotation and genome-wide methylation patterns for the *H*. *parasuis* non-virulent D74 strain and for the highly virulent Nagasaki strain. This collective information will enable reliable one-to-one assignment of specific genes of interest and subsequent evaluation of predicted protein structures. Highlights of the information gained from this study include the sequence and annotation of a plasmid harbored by strain D74 that shares a high degree of similarity to other plasmids harbored by members of the *Pasteurellaceae* family, which could prove useful in future allelic replacement and/or functional genomic studies. Evaluation of the virulence-associated genes contained within the genomes of D74 and Nagasaki led to the discovery of a large number of TA systems, primarily Type II TA families, within both genomes. Five predicted hemolysins were identified as unique to Nagasaki and seven putative contact-dependent growth inhibition toxin proteins were identified only in strain D74. Assessment of all potential *vtaA* genes revealed thirteen present in the Nagasaki genome and three in the D74 genome. Subsequent evaluation of the predicted protein structure revealed that none of the D74 VtaA proteins contain a collagen triple helix repeat domain and a much larger predicted amino acid size for two D74 VtaA proteins compared to any predicted Nagasaki VtaA protein. Fifteen methylation sequence motifs were identified in D74 and fourteen methylation sequence motifs were identified in Nagasaki using SMRT sequencing analysis. Only one of the methylation sequence motif was observed in both strains highlighting the diversity between D74 and Nagasaki. Subsequent analysis also revealed diversity in the restriction-modification systems harbored by D74 and Nagasaki. Our hope is that the assembly and annotation of these genomes, coupled with the comparative genomic analyses reported in this study, will aid in the identification of genetic elements that underlie and influence phenotypic differences between these isolates. Together, this information can support future research and the development of vaccines with improved efficacy towards *H*. *parasuis* in swine to decrease the prevalence and disease burden caused by this pathogen.

## Supporting information

S1 Table D74 CDS list. (XLSX)

S2 Table Nagasaki CDS list. (XLSX)

S3 Table AMR MIC data. (XLSX)

S4 Table D74 capsule genes. (DOCX)

S5 Table Nagasaki capsule genes. (DOCX)

S6 Table D74 RM gene SSRs. (XLSX)

S7 Table Nagasaki RM gene SSRs. (XLSX)

S1 File D74 annotations. (GB)

S2 File. Nagasaki annotations (GB)

S3 File. pD74 annotations (GB)
