# Supplementary material for: Comparative genomic and methylome analysis of non-virulent D74 and virulent Nagasaki Haemophilus parasuis isolates
Source: PLoS One. 2018 Nov 1;13(11):e0205700. doi: 10.1371/journal.pone.0205700 (PMC6211672; doi:10.1371/journal.pone.0205700)
Supplement: S4 Table — (DOCX) [file pone.0205700.s004.docx]

**S4 Table.** Nucleotide and amino acid differences in *H. parasuis* D74 capsule genes reported here compared to the corresponding capsule sequence reported by Howell et al. [10].

| **Howell et al. [10] Accession** | **Howell et al. [10] Name** | **D74 locus_tag** | **D74 Name** | **% Nucleotide Identity** | **Nucleotide Differences** | **% Protein Identity** | **Amino Acid Differences** | **Total Predicted Amino Acids Changes^a^** |
| --- | --- | --- | --- | --- | --- | --- | --- | --- |
| KC795423 | *funA9* | A2U20_03720 | *funA* | 99.8 | 1 | 100 | 0 |  |
| KC795425 | *neuA9* | A2U20_03725 | *neuA3* | 98.8 | 8 | 98.7 | 3 | K61R; I65T; P66A |
| KC795426 | *wzx9* | A2U20_03730 | *wzx2* | 98.2 | 21 | 98.2 | 7 | V176A; I180V; A187V; S192N; R278H; P291S; R373S |
| KC795427 | *astA9* | A2U20_03735 | *astA***^b^** | 98 | 20 | 97.3 | 9 | N98_H99insHI**^d^**; Q134R; S157A; V177I; V183I; I189V; S195N; V2056M |
| KC795428 | *funP9* | A2U20_03740 | *funP* | 99.5 | 4 | 99.6 | 1 | E29D |
| KC795429 | *funV9* | A2U20_03745 | *funV* | 99.9 | 1 | 100 | 0 |  |
| KC795430 | *gltL9* | A2U20_03750 | *gltL***^c^** | 100 | 0 | 100 / 99.7 | 9 | M1_K8del**^d^**; V9M |
| KC795431 | *gltM9* | A2U20_03755 | *gltM* | 100 | 0 | 100 | 0 |  |
| KC795432 | *ndeB9* | A2U20_03760 | *ndeB* | 99.9 | 1 | 99.6 | 1 | A131V |
|  |  | A2U20_03765 |  |  |  |  |  |  |
| KC795433 | *pilT9* | A2U20_03770 | *pilT* | 99.7 | 1 | 100 | 0 |  |
| KC795434 | *cap5M9* | A2U20_03775 | *cap5M* | 99.8 | 1 | 99.5 | 1 | L30F |
|  |  | A2U20_03780 |  |  |  |  |  |  |
| KC795435 | *capD9* | A2U20_03785 | *capD* | 99.6 | 7 | 99.5 | 3 | N138K; G293A; G448D |
| KC795436 | *wza9* | A2U20_03790 | *wza* | 99.7 | 3 | 99.7 | 1 | D33N |
| KC795438 | *wzb9* | A2U20_03795 | *wzb* | 100 | 0 | 100 | 0 |  |
| KC795439 | *wzs9* | A2U20_03800 | *wzs* | 99.6 | 8 | 99.3 | 5 | E3K; L27I; V230A; V613A; V622A |
| KC795440 | *iscR9* | A2U20_03805 | *iscR* | 99.3 | 3 | 99.3 | 1 | V89L |

^a^Residue numbers based on Howell et al. [10] sequence as reference.

^b^*astA* (A2U20_03735) predicted to encode a longer amino acid sequence compared to Howell et al. [10] sequence with 6 nucleotide insertions corresponding to 2 amino acid insertions.

^c^*gltL* (A2U20_03755) predicted to encode a shorter amino acid sequence compared to Howell et al. [10] sequence due to a different predicted start codon.

^d^Depicted using Human Genome Variant Society (HGVS) nomenclature [44].
