# Supplementary material for: Comparative genomic and methylome analysis of non-virulent D74 and virulent Nagasaki Haemophilus parasuis isolates
Source: PLoS One. 2018 Nov 1;13(11):e0205700. doi: 10.1371/journal.pone.0205700 (PMC6211672; doi:10.1371/journal.pone.0205700)
Supplement: S5 Table — (DOCX) [file pone.0205700.s005.docx]

**S5 Table.** Nucleotide and amino acid differences in *H. parasuis* Nagasaki capsule genes reported here compared to the corresponding capsule sequence reported by Howell et al. [10].

| **Howell et al. [10] Accession** | **Howell et al. [10] Name** | **Nagasaki locus_tag** | **Nagasaki Name** | **% Nucleotide Identity** | **Nucleotide Differences** | **% Protein Identity** | **Amino Acid Differences** | **Total Predicted Amino Acids Changes^a^** |
| --- | --- | --- | --- | --- | --- | --- | --- | --- |
| KC795336 | *funA5* | A2U21_07940 | *funA***^b^** | 95.3 | 22 / 111 | 96.8 | 5 / 37 | M1ext-37**^d^**; A2V; A6V; D27V; S28N; V138I |
| KC795337 | *neuA5* | A2U21_07935 | *neuA* | 97.7 | 16 | 99.6 | 1 | S165Q |
| KC795338 | *wzx5* | A2U21_07930 | *wzx* | 98.2 | 22 | 98.5 | 6 | V95A; L150F; I275T; Q280E; A321V; F391Y |
| KC795339 | *lsgB5* | A2U21_07925 | *lsgB* | 99.6 | 4 | 99.1 | 3 | Y28H; F74V; H150Q |
| KC795340 | *funK5* | A2U21_07920 | *funK* | 99.3 | 7 | 97.9 | 7 | V87I; G90S; A117T; S246F; K303E; G304E; I312V |
| KC795341 | *wcwK5* | A2U21_07915 | *wcwK* | 99.8 | 2 | 99.7 | 1 | V49A |
| KC795342 | *wcfQ5* | A2U21_07910 | *wcfQ* | 95.1 | 40 | 95.6 | 12 | E43K; I58V; S61T; I64T; F106S; D131G; F132S; N194K; Y262C; R269N; F270N; L271F |
| KC795343 | *wbgX5* | A2U21_07905 | *wbgX***^c^** | 97.3 | 35 | 98.2 | 8 | M1del**^d^**; K2M; E3N; F4Y; T177A; S343N; P344A; A349I |
| KC795344 | *wbgY5* | A2U21_07900 | *wbgY* | 99.2 | 5 | 99.5 | 1 | V187I |
| KC795345 | *capD5* | A2U21_07895 | *capD* | 99.6 | 7 | 99.4 | 4 | F89S; H340Y; S431G; V578I |
| KC795346 | *wza5* | A2U21_07890 | *wza***^d^** | 99.2 | 9 | 99.2 | 3 | A122T; N311_E312insN**^d^**; I316T |
| KC795348 | *wzb5* | A2U21_07885 | *wzb* | 100 | 0 | 100 | 0 |  |
| KC795349 | *wzs5* | A2U21_07880 | *wzs* | 99.8 | 4 | 99.6 | 3 | S75A; S420L; G562E |
| KC795350 | *iscR5* | A2U21_07875 | *iscR* | 99.6 | 2 | 100 | 0 |  |

^a^Residue numbers based on Howell et al. [10] sequence as reference.

^b^*funA* (A2U21_07940) predicted to encode a longer amino acid sequence compared to Howell et al. [10] sequence due to a different predicted start codon and 22 nucleotide differences corresponding to 5 amino acid within the shared region. FunA is predicted to contain with 37 additional amino acids on the N-terminus.

^c^*wbgX* (A2U21_07905) predicted to encode a shorter amino acid sequence compared to Howell et al. [10] sequence due to a different predicted start codon.

^d^*wza* (A2U21_07890) predicted to encode a longer amino acid sequence compared to Howell et al. [10] sequence with 3 nucleotide insertions corresponding to 1 amino acid insertion.

^d^Depicted using HGVS nomenclature [44].
